# Bioinformatic Analysis of Temporal and Spatial Proteome Alternations During Infections

**DOI:** 10.3389/fgene.2021.667936

**Published:** 2021-07-02

**Authors:** Matineh Rahmatbakhsh, Alla Gagarinova, Mohan Babu

**Affiliations:** ^1^Department of Biochemistry, University of Regina, Regina, SK, Canada; ^2^Department of Biochemistry, Microbiology, & Immunology, University of Saskatchewan, Saskatoon, SK, Canada

**Keywords:** temporal proteomics, spatial proteomics, host-pathogen interactions, clustering, principal component analysis, self-organizing maps, data imputation, normalization

## Abstract

Microbial pathogens have evolved numerous mechanisms to hijack host’s systems, thus causing disease. This is mediated by alterations in the combined host-pathogen proteome in time and space. Mass spectrometry-based proteomics approaches have been developed and tailored to map disease progression. The result is complex multidimensional data that pose numerous analytic challenges for downstream interpretation. However, a systematic review of approaches for the downstream analysis of such data has been lacking in the field. In this review, we detail the steps of a typical temporal and spatial analysis, including data pre-processing steps (i.e., quality control, data normalization, the imputation of missing values, and dimensionality reduction), different statistical and machine learning approaches, validation, interpretation, and the extraction of biological information from mass spectrometry data. We also discuss current best practices for these steps based on a collection of independent studies to guide users in selecting the most suitable strategies for their dataset and analysis objectives. Moreover, we also compiled the list of commonly used R software packages for each step of the analysis. These could be easily integrated into one’s analysis pipeline. Furthermore, we guide readers through various analysis steps by applying these workflows to mock and host-pathogen interaction data from public datasets. The workflows presented in this review will serve as an introduction for data analysis novices, while also helping established users update their data analysis pipelines. We conclude the review by discussing future directions and developments in temporal and spatial proteomics and data analysis approaches. Data analysis codes, prepared for this review are available from https://github.com/BabuLab-UofR/TempSpac, where guidelines and sample datasets are also offered for testing purposes.

## Introduction

Intracellular pathogens, including viruses, bacteria (Auweter et al., 2011; Schweppe et al., 2015; Lopez et al., 2016), parasites, and fungi (Iyer et al., 2007; Gilbert et al., 2015; May and Casadevall, 2018; Eisenreich et al., 2019), cause numerous deaths and impose staggering healthcare costs (Kamaruzzaman et al., 2017). Spatially and temporally intricate progression of interplay between the host and the pathogen results in disease. This interplay in host-pathogen interactions (HPI) is highly complex and dynamic. Although mechanistic details vary, all intracellular pathogens need to enter the host cell, avoid or exploit the host’s defense mechanisms, and exploit the host’s resources (e.g., lipids, proteins, and metabolites) for replication and spread to neighboring cells (Jean Beltran et al., 2016). Studies of HPI-dependent alterations to the host cell’s proteome not only reveal components required for pathogenesis but also provide critical insights into host processes (e.g., see Alto and Orth, 2012; Jo, 2019 and references therein).

A major key to combatting intracellular pathogens lies in the understanding of how they hijack host systems. This, in turn, requires the mapping the spatial and temporal proteome changes underlying disease progression. These may include changes in protein abundance, interactions, localizations, or posttranslational modifications (Ribet and Cossart, 2010; Gagarinova et al., 2017; Scott and Hartland, 2017; Grishin et al., 2021). For example, Weekes et al. (2014) mapped proteome changes occurring during the course of the human cytomegalovirus infection, thereby identifying key temporal changes and potential new targets for antiviral therapies. Likewise, pathogens actively regulate organelle dynamics (Auweter et al., 2011; Schweppe et al., 2015; Lopez et al., 2016; Selkrig et al., 2020).

Technological advances in mass spectrometry (MS)-based proteomics and bioinformatics allow achieving temporal and spatial resolution of the infection process at previously unseen levels (e.g., see Lopez et al., 2016; Jean Beltran et al., 2017; Selkrig et al., 2020 and references therein). See Kumar and Mann (2009) and Jean Beltran et al. (2016) for an overview of quantitative proteomic approaches and relevant computational methods. The typical output of a quantitative MS experiment that maps temporal and/or spatial changes during an infection includes highly complex, multi-dimensional data matrices with protein abundances across space or time represented by ion intensities or spectral counts, depending on the MS approach. Such data are challenging to analyze and interpret. However, a review covering such downstream analyses has been lacking. We present frameworks for the analysis of temporal (section “Temporal Analysis of Proteome Changes in an Infected Cell”) and spatial (section “Exploring Subcellular Proteome Organization During Infection”) proteomic data from HPI studies, focusing on specific examples and robust methods adapted from statistics and machine learning. We also discuss measures for validating the results and describe how these frameworks can be implemented in R programming language, suggesting appropriate software packages where applicable (all packages are summarized in Table 1). Moreover, we combined useful functions into workflows in R programming language. These are available at https://github.com/BabuLab-UofR/TempSpac. The workflows we discuss and present can be used to extract biological meaning from MS data to model disease progression and drive therapeutics discovery. Although examples in this review focus on intracellular pathogens, the same pipelines can be used, e.g., in the analysis of genetic or environment-induced disease.

**TABLE 1:** List of packages and useful functions.

Sections	Packages	Descriptions and useful functions	References
Unsupervised clustering (section “Clustering Analyses”)	stats	hclust() = agglomerative hierarchical clustering	RStudio Team, 2020
		cuttree() = control the number of generated clusters	
		kmeans() = *K-*mean clustering	
	cluster	diana() = divisive hierarchical clustering	Maechler et al., 2019
		agnes() = agglomerative hierarchical clustering	
		fanny() = fuzzy clustering	
	hybridHclust	mutualCluster() = mutual cluster	Chipman and Tibshirani, 2006
	Mfuzz	mfuzz() = fuzzy clustering	Kumar and Futschik, 2007
	e1071	cmeans() = fuzzy clustering	Meyer et al., 2020
	ppclust	fcm() = fuzzy clustering	Cebeci, 2019
	pracma	Kmeanspp() = *k*-means++ clustering algorithm	Borchers, 2019
	Kohonen	som() = self-organizing map (som) clustering	Wehrens and Kruisselbrink, 2019
	clValid	clValid() = clMethods argument specifies clustering methods e.g., “hierarchical,” “kmeans,” etc.	Brock et al., 2011
		clValid() = validation argument specifies validation measures e.g., “biological,” “internal,” etc.	
	ClusterR	external_validation() = external measures	Mouselimis et al., 2020
	factoextra	fviz_nbclust() = define optimal number of clusters	Kassambara and Mundt, 2020
	ggfortify	autoplot() = output appropriate plots based on the type of unsupervised clustering	Tang et al., 2016
Supervised clustering (section “Predicting Protein Localizations in Each Condition”)	pRoloc	knnClassification() = *k*-nearest neighbors (*k-*NN) algorithm	Gatto et al., 2014b
		knnOptimisation() = classification parameter optimization for *k-*NN	
		svmClassification() = support vector machine (svm) algorithm	
		svmOptimisation() = classification parameter optimization for svm	
		nnetClassification() = neural net (nnet) algorithm	
		nnetOptimisation() = classification parameter optimization for nnet	
		nbClassification() = naïve bayes (nb) algorithm	
		nbOptimisation() = classification parameter optimization for nb	
	caret	train() = to fit a model	Kuhn et al., 2020
		trainControl() = control parameters for training	
		predict() = predict probability scores	
Data normalization (sections “Quantitative Temporal Data Visualization, Preprocessing, and Quality Control” and “Data Normalization”)	edgeR	caclNormFactors() = method argument specifies the type of normalization (e.g., TMM)	Robinson et al., 2010
	DESeq2	estimateSizeFactors() = compute scaling factor for normalization by RLE method	Love et al., 2014
		counts() = retrieve normalized matrix counts	
	DEP	normalize_vsn() = perform normalization using that variance stabilization normalization (Vsn)	Zhang et al., 2018
	vsn	Justvsn() = perform normalization using variance stabilization normalization (Vsn)	Huber et al., 2002
	MASS	lm() = perform linear regression	Venables and Ripley, 2002
Missing value imputation (sections “Quantitative Temporal Data Visualization, Preprocessing, and Quality Control” and “Missing Value Imputation”)	pcaMethods	llsImpute() = perform imputation of missing value using local least squares approach (LLS)	Stacklies et al., 2007
	bnstruct	knn.impute() = perform imputation of a missing value using *k-*NN	Franzin et al., 2017
	DEP	impute() = missing value imputation; fun argument specifies imputation method e.g., *k*-NN, “QRILC,” etc.	Zhang et al., 2018
	mice	mice() = perform imputation of missing values using mice	Van Buuren and Groothuis-Oudshoorn, 2010
	MSstats	MBimpute() = impute missing values	Choi et al., 2014
Differential expression analysis (Section “Statistical Analysis of Quantitative Temporal Proteomics Data”)	MSstats	detect differentially expressed proteins in both label-free and labeling-based experimental approaches	Choi et al., 2014
	MSstatsTMT	detect differentially expressed proteins in experiments with isobaric labeling	Huang et al., 2020
	DEP	detect differentially expressed proteins in experiments from both label-free and labeling-based experimental approaches	Zhang et al., 2018
	limma	detect differentially expressed proteins when sample sizes are small (<10)	Ritchie et al., 2015
	DESeq2	detect differentially expressed proteins when sample sizes are small (<10)	Love et al., 2014
	edgeR	detect differentially expressed proteins when sample sizes are small (<10)	Robinson et al., 2010
Dimensionality-reduction techniques (sections “Quantitative Temporal Data Visualization, Preprocessing, and Quality Control” and “Dimensionality Reduction Tools for Visualizing Organellar Map”)	tnse	tnse() = t-SNE dimensionality reduction	Donaldson, 2016
	umap	umap() = UMAP dimensionality reduction	Konopka, 2020
	stats	prcomp() = PCA dimensionality reduction	RStudio Team, 2020
		princomp() = PCA dimensionality reduction	

*Additional scripts, written for this review are provided at https://github.com/BabuLab-UofR/TempSpac.*

## Temporal Analysis of Proteome Changes in an Infected Cell

Several temporal studies employed quantitative whole-cell proteomics in order to quantify changes occurring during the course of a productive viral infection, helping elucidate HPI mechanisms, immune responses, and mechanisms of immune system evasion by the pathogen (Weekes et al., 2014; Greenwood et al., 2016; Clements et al., 2017; Caller et al., 2019; Soday et al., 2019). For instance, Soday et al. (2019) achieved extensive host and viral proteome coverage, and their downstream analyses revealed multiple pathways dysregulated in response to Vaccinia virus infection. These included antiviral factors, collagens, and interferon-stimulated genes (e.g., IFITM3) (Soday et al., 2019).

Moreover, quantitative temporal whole-cell proteomics in the presence and absence of a specific viral protein has been used to elucidate how the selected viral protein contributes to disease (e.g., Lapek et al., 2017; Greenwood et al., 2019). For instance, Lapek and colleagues reported that Vpr, a human immunodeficiency virus protein, mediated the modulation of serine/arginine-rich protein-specific kinases, spindle and centromere proteins, and others (Lapek et al., 2017). Thus, a potential role for Vpr in RNA splicing *via* serine/arginine-rich protein-specific kinases has been suggested (Lapek et al., 2017). Although other approaches would need to be employed to distinguish direct vs. indirect effects of specific viral proteins, this approach provides a framework for dissecting the activities of specific proteins in pathogenesis.

Although whole-cell temporal quantitative proteomic analyses reveal key pathways and proteins affected by infection, they do not contain spatial information about protein dynamics within subcellular compartments, which is essential to understand the organization of proteome upon infection and the underlying mechanisms. Organelle temporal proteomics reveal dynamic changes on sub-cellular level with higher resolution than whole-cell proteomics due to better ability to detect low-abundance proteins. Moreover, temporal proteomic data from whole cells and subcellular fractions can be integrated in order to compare the total abundance of a given protein in whole-cell lysate vs. a specific organelle to better understand disease progression and pathogenesis strategies. For instance, Weekes et al. (2014) quantified temporal human cytomegalovirus-induced changes both in whole-cell lysates and at cell surface. The results indicated that human cytomegalovirus infection resulted in rapid depletion of CD155 (poliovirus receptor, PVR) from the cell surface at the same time as the total amount of CD155 in the whole cell increased (Weekes et al., 2014). CD155 is a ligand involved in the activation of natural killer cell-mediated immunity against human cytomegalovirus (Tomasec et al., 2005). Therefore, sequestration of CD155 may be one of the pathogenesis strategies of human cytomegalovirus (Weekes et al., 2014).

**FIGURE 1:**
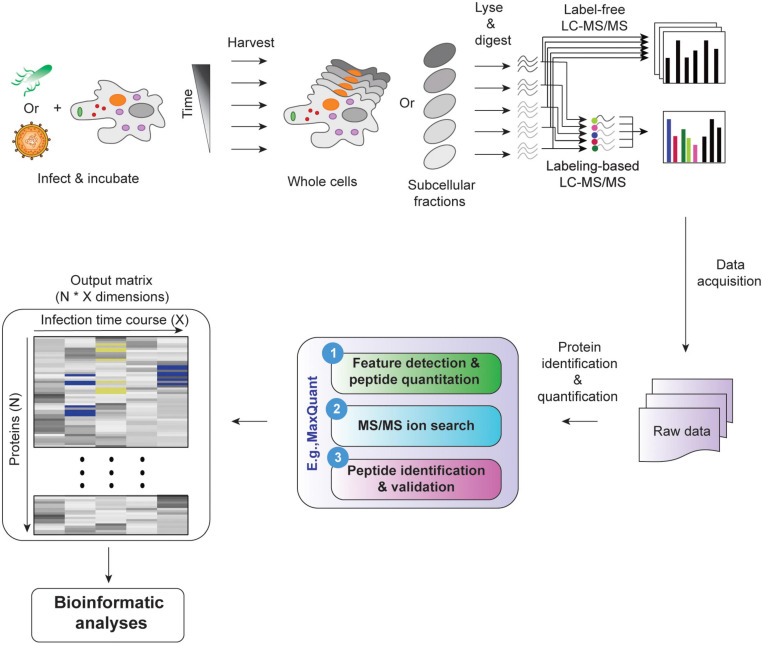
Temporal MS-based proteomic HPI studies combine advanced experimental platforms, instrumentation, and software to study alterations in protein abundances over the course of an infection. Following infection, cells (whole-cell temporal proteomics) or organelles (organelle temporal proteomics) are harvested at different time points post-infection, followed by lysis and proteolysis. The resulting peptides are then analyzed by label-free or multiplexed label-based (e.g., TMT or SILAC) quantitative MS (for a review, see Jean Beltran et al., 2017). Software, such as MaxQuant can be used to process the data and generate a multidimensional matrix that contains information about protein identities (N) and their relative abundances across X time points in infected vs. uninfected samples. This is then used to assess temporal changes in cell proteome during infection.

**FIGURE 2:**
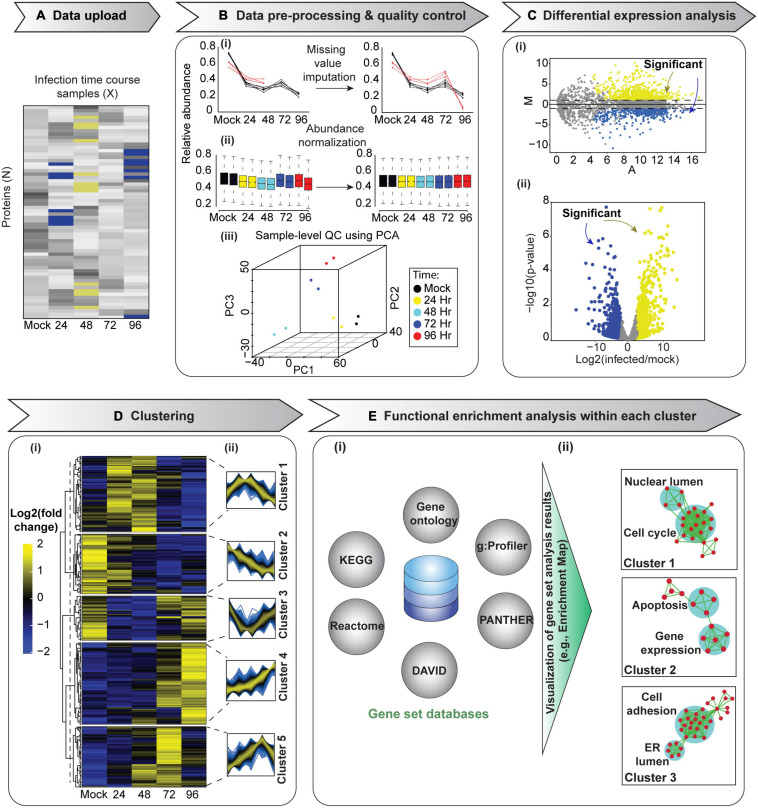
A schematic of temporal proteomic data analysis pipeline. After upload **(A)**, data pre-processing and quality control steps are performed **(B)**. These include the imputation of missing values (i), normalization (ii), and sample-level quality control, e.g., using principal component analysis (PCA, iii). **(C)** Differential expression analysis can then be performed to identify proteins with significantly altered expression between states, for example, different relative (infected/mock) expression between time points. Results can be visualized in (i) an MA plot [log fold change, M, vs. log of the mean expression level between conditions, (**A**)] or in (ii) a volcano plot. Subsequently, clustering analysis can be performed to group proteins with similar temporal expression patterns **(D)**. Here, we applied hierarchical clustering, which is displayed in conjunction with a heat map visualization of the clustered data. The dendrogram was cut at the level indicated by the dashed line to yield five clusters. (ii) Each cluster’s temporal profiles can be visualized in a simple plot with relative abundances (y-axis) of proteins within each cluster across all time points (x-axis). Finally, functional enrichment analysis of each of these clusters could provide information on pathways and cellular processes rewired in response to an infection **(E)**. (i) Groups for the analysis can be derived from annotations (e.g., gene ontology; Gene Ontology Consortium, 2004), a pathway database (e.g., KEGG; Kanehisa et al., 2012, Reactome; Croft et al., 2011), or a combination of these (e.g., DAVID; Sherman et al., 2007, PANTHER; Mi et al., 2013, and g:Profiler; Reimand et al., 2007). (ii) Since many inter-dependent gene sets may be enriched, organizing results into a network, e.g., by means of EnrichmentMap (Merico et al., 2010) can be useful. Here, gene sets that share many proteins are grouped together, thereby offering an intuitive visualization of the results. We used 1,000 randomly selected proteins from Weekes et al. (2014) experiment 1 to exemplify data analysis steps.

In a temporal proteomic HPI study, infected and uninfected cells or organelles are collected and processed for quantitative MS (Figure 1). The MS data are then analyzed by specialized software, such as MaxQuant (Cox and Mann, 2008; Chen et al., 2020). As a result, multidimensional data with information about protein identities and abundances in infected vs. uninfected cells across time are obtained. Sections “Quantitative Temporal Data Visualization, Preprocessing, and Quality Control”–“Evaluation Measures for Temporal Clustering” present a robust workflow for the downstream analysis of such data (Figure 2).

### Quantitative Temporal Data Visualization, Preprocessing, and Quality Control

The data generated by temporal profiling can be represented in a matrix format with features (i.e., proteins) and different time points along rows and columns, respectively Figure 2A. The first step in data analysis is to check data quality by using a set of metrics or through visualization. Here, first, data distribution, variation, and other descriptive statistics are assessed (e.g., using box plot, line chart, histogram, and density plot). There is a handy multipurpose function called summary() in R language (RStudio Team, 2020) that provides descriptive statistics for each variable or column and reports the number of missing values in the dataset. One can also directly visualize the temporal profile of each protein by plotting its abundance or relative intensity across different time points (Figure 2Bi). Such plots help detect misidentified features (i.e., proteins) with inconsistent temporal quantitative profiles or missing values.

Data preprocessing includes the imputation of missing values and the normalization of the data (Figures 2Bi,ii). Data preprocessing is essential in the analysis of quantitative proteomics data (Karpievitch et al., 2012). For example, data may be missing for low-abundance proteins (Karpievitch et al., 2012). These missing values may be removed or imputed. The easiest way to impute a missing value in temporal data is to fit a curve to the incomplete temporal data, and then missing abundance could be imputed based on the fitted values in the curve. However, this method has a disadvantage of introducing potential artifacts as the time series will be constrained to follow the fitted curve (Du et al., 2008). It is usually assumed that there is no dramatic change in abundance values between nearest time points; therefore, abundance values at the nearby time points could be used to impute each missing abundance value (Du et al., 2008). The imputation of missing values in time-series datasets and the effect of different imputation methods on the resulting inferences have been extensively studied for microarray analyses. The same methods can be applied to the temporal proteomic data. For instance, Chiu et al. (2013) evaluated the performance of nine different imputation algorithms. LLS-like algorithms [local least squares (LLS), iterative local-least-squares (ILLS), and sequential local-least-squares (SLLS)] outperformed other algorithms for time-series datasets. The LLS imputation algorithm (Kim et al., 2005) first identifies genes similar to the gene with a missing value by applying a distance measure (e.g., Euclidean Distance or Pearson correlation coefficient). Then, the missing value is estimated by representing the target gene as a linear combination of similar genes. This method is implemented as llsImpute() function in the pcaMethods Bioconductor package (Stacklies et al., 2007) in the R environment.

Data normalization aims to eliminate systematic biases to allow statistical inferences (Karpievitch et al., 2012). MS-based data are typically biased due to a number of factors. One of them is inadequate normalization before LC/MS (Wisìniewski and Mann, 2016; Chen et al., 2020). Therefore, selecting an appropriate normalization method is essential. The concept of data normalization in transcriptomics and proteomics has been explored extensively (see Karpievitch et al., 2012; Välikangas et al., 2018; Chen et al., 2020), albeit not in the context of temporal quantitative proteomics data. The dynamic pattern of protein abundances is a key measure in a time-course study. Different time points can be normalized against the same reference sample, included in each MS run (Du et al., 2008; Li et al., 2018; Nusinow and Gygi, 2020). For instance, the relative intensity for each protein can be normalized to the sum or average of all protein intensities in the reference sample (Nusinow and Gygi, 2020). However, in this case, biases may persist (see Murie et al., 2018 and references therein). Therefore, data may instead be adjusted for protein loading across all channels/samples by normalizing the intensity of each particular protein to mean, median, or overall sum of all intensities across all channels/samples (Jean Beltran et al., 2016; Soday et al., 2019; Nusinow and Gygi, 2020). For instance, the “total count” approach aims to equalize protein loading across all channels/samples using a normalization factor, which is the sum of all intensities (or spectral counts) in a given MS run.

However, the application of a more sophisticated normalization method would be welcome when comparing multiple experimental conditions (e.g., “infected” vs. “uninfected”) to identify differentially expressed proteins. When multiple conditions are included in the analysis, the “total count” approach can bias the results to be skewed toward one experimental condition if proteins between biological conditions are disproportionately represented. The TMM [“weighted trimmed mean of M-values (i.e., log-intensity ratios)”] normalization may provide a more robust approach to calculate a normalization factor (Robinson and Oshlack, 2010). The TMM normalization method is based on the hypothesis that most features (e.g., proteins) are not differentially expressed. It selects one sample as a reference and computes a TMM normalization factor for the remaining non-reference samples (Robinson and Oshlack, 2010). This approach is employed in the edgeR Bioconductor package as the default normalization method (Robinson et al., 2010). Like TMM, “relative log expression” (RLE) normalization assumes that most genes or proteins are not differentially expressed. For a given sample, the RLE scaling factor is determined by dividing the observed counts of each feature (e.g., protein) by its geometric mean across all samples. This normalization method is included in the DESeq and DESeq2 Bioconductor packages (Anders and Huber, 2010; Love et al., 2014). The TMM and RLE have been thoroughly investigated. They effectively eliminate biases in RNA sequencing data due to unequal library size (Dillies et al., 2013), as well as in proteomic data (Branson and Freitas, 2016). They have been developed for spectral count data but should be applicable to intensity measurements as well. Indeed, normalization methods applicable to count data (e.g., RNA sequencing, peptide counts) have been successfully used with proteomic intensity measurements (Gatto et al., 2014a). Note that both, spectral counts and ion intensity values, should be log_2_ transformed [i.e., log_2_(value+1)] to normalize their distributions prior to using DESeq2 Bioconductor package (Huang et al., 2020). Log2 normalization is included in edgeR package, so it is not required prior to TMM normalization by means of edgeR package.

The selection of the optimum normalization approach is greatly dependent on the experimental design. The efficiency of each normalization method can be evaluated using box (Figure 2Bii) or MA (log fold change, M, vs. log of the mean expression level between conditions, A) plots (Figure 2Ci). The MA plots are a convenient pairwise representation of conditional data commonly employed in proteomics (Breitwieser et al., 2011; Gatto and Lilley, 2012). After appropriate normalization, most data points in an MA plot will be clustered around *M* = 0, while points away from *M* = 0 identify differentially expressed proteins (Figure 2Ci; Chen et al., 2020). Moreover, if data preprocessing and normalization were effective, patterns are expected to emerge in subsequent analyses steps (see below).

Following data normalization, visualizing entire dataset in one figure is often needed to evaluate data quality and structure. Principal component analysis (PCA) allows the visualization of high-dimensional data in a reduced set of dimensions, generally in two or three, while retaining as much of the initial information as possible (Figure 2Biii). It does this by transforming correlated variables into fewer uncorrelated variables called principal components (PCs), which are then arranged according to the amount of variability described in each component. The first PC accounts for the most variation in the original data, the second PC accounts for most of the residual variation, etc. (Lever et al., 2017). Each succeeding component represents as much of the remaining variability as possible: it represents more variability than the PC after it and less than the one before, with the last PCs containing mostly noise and very little information. Therefore, well-structured datasets could be visualized as a projection of the first two or three PCs in a 2-D or a 3-D plot (Figure 2Biii); such a plot is a simple yet informative depiction of the whole dataset. If data structure exists, proteins with similar informative characteristics (e.g., with similar functional annotations) will be grouped together in a 2-D or a 3-D plot, and dissimilar groups (i.e., with divergent unrelated functions) would be located far from each other. However, if normalization and the correction of technical artifacts were insufficient, proteins from different technical replicates may form distinct clusters instead.

PCA is an unsupervised machine learning technique, meaning samples are not associated with a class label. Instead, a pattern in the graphical representation results from similarities in attributes. The use of an unsupervised PCA clustering without external information is an efficient quality control and data analysis approach. For example, PCA helps detect the presence of batch effects and outliers: it will reveal how replicates cluster together. If, for example, a batch effect is apparent, normalization was not sufficient and needs to be adjusted. Furthermore, PCA allows an unbiased representation of the main patterns in the data before biologically relevant parameters are mapped (e.g., subjects clustering in line with predicted treatment group) (Figure 2Biii). If no structure is evident, one would not expect well-defined temporal clusters, and hence, the statistical inferences from such data will be challenging. PCA has been widely applied in the analysis of proteomic data (Purohit and Rocke, 2003; Hou et al., 2017; Itzhak et al., 2019; Santana-Codina et al., 2020), as well as in temporal proteomic HPI studies (Diamond et al., 2010; Weekes et al., 2014; Greenwood et al., 2019). However, if more than three PCs are required to describe at least 60% of variance in the data (Hair et al., 2009), other dimensionality reduction tools must be used to visualize the dataset (see sections “Self-Organizing Map” and “Dimensionality Reduction Tools for Visualizing Organellar Map”). The stats (RStudio Team, 2020) package in R has two built-in functions including prcomp() and princomp() that can be used to perform PCA analysis. Other packages, such as factoextra (Kassambara and Mundt, 2020) and ggfortify (Tang et al., 2016) also offer users ggplot2-based elegant visualization capabilities.

### Statistical Analysis of Quantitative Temporal Proteomics Data

Following data pre-processing, the next step is to accurately identify proteins with significantly different expression between samples. The outcome of differential analysis is often visualized *via* MA or Volcano plots (Figures 2Ci,ii; Kucukural et al., 2019). The identification of differentially expressed proteins across time points can help pinpoint predictive factors or biomarkers for disease. A traditional *t*-test; its nonparametric equivalent, the Wilcoxon test; or the analysis of variance (ANOVA) are the most standard approaches to delineating significantly altered protein abundances (Diamond et al., 2010; Weekes et al., 2014; Itzhak et al., 2019; Soday et al., 2019). Another method to identify differentially expressed proteins from both label-free and labeling-based experimental approaches is offered by MSstats. MSstats is a Bioconductor package in R environment that relies on linear mixed models. It takes the data input, detects study design, and, based on the design, fits an appropriate linear mixed model to discover differentially expressed proteins (Choi et al., 2014). The MSstatsTMT Bioconductor R package is also available for applying Empirical Bayes procedure in R to detect differentially expressed proteins in experiments with isobaric labeling (Huang et al., 2020). Furthermore, the DEP Bioconductor package in R can be used to detect differentially expressed proteins from both label-free and labeling-based experimental approaches (Zhang et al., 2018).

However, in a shotgun discovery-based proteomics experiment, sample sizes are often small (<10), which results in ambiguity in the estimation of variability. This, in turn, may result in a non-significant *p*-value for proteins with a substantial fold change due to large sample variance and a significant *p*-value for proteins with small fold change due to small sample variance. To overcome this issue, Kammers et al. (2015) improved the detection of differentially expressed proteins by applying moderated *t*-test statistics from an empirical Bayes method called “Linear Models for Microarray Data” (limma). Limma adjusts variances toward a pooled estimate based on all sample data. This results in a more powerful detection of differences, especially for experiments with a relatively small sample size (Smyth, 2004) while still allowing a distribution of variances (Kammers et al., 2015). Limma is available as a Bioconductor package in R (Ritchie et al., 2015), and has been frequently used for proteomics data analyses (Brusniak et al., 2008; Margolin et al., 2009; Ting et al., 2009; Schwämmle et al., 2013; Zhao et al., 2013; Greenwood et al., 2019). DESeq (Anders and Huber, 2010) and edgeR (Robinson et al., 2010) are Bioconductor R packages suitable for differential expression analyses with relatively small proteomic datasets (Branson and Freitas, 2016). Differential expression can likewise be assessed by the generalized linear mixed-effects model (GLMM) (Choi et al., 2008), linear mixed-effects model (Hill et al., 2008), or quasi-likelihood modeling generalized linear model (GLM) (Li et al., 2019).

Isobaric-labeling based MS approaches are suffering from “ratio compression,” i.e., the estimated protein abundance ratio level across samples is typically underestimated. This occurs due to interference with quantification from co-fragmented peptides (Rauniyar and Yates, 2014). This undermines the ability of isobaric labeling to be genuinely quantitative (Li et al., 2019). Methods exist to correct for “ratio compression” (Savitski et al., 2013), but detailed discussion of the topic is outside the scope of this manuscript. Several statistical models have been developed to accurately measure and correct the technical variability of isobaric-labeling based MS approaches (Huang et al., 2020). Some of these models depend on either prior knowledge or separate experiments to evaluate noise levels in the data (Zhang et al., 2010; Breitwieser et al., 2011; Zhou et al., 2012, 2014a). “Model-based analysis of proteomic data” (MAP) is also available for label-based experimental workflows. Unlike earlier algorithms that required technical replicates to determine experimental error and identify proteins with significantly altered expression, MAP uses regression analysis to calculate local error. These error estimates are then employed in the detection of proteins with significantly altered expression without the need for technical replicates to model technical and systematic errors (Li et al., 2019). In comparative analyses, MAP outperformed other, replicate-based algorithms (Zhang et al., 2010). It is therefore the currently preferred tool for the analysis of label-based proteomics data.

For all statistical tests above, it is essential to correct for multiple hypothesis testing, as many tests are conducted simultaneously. This can be done by controlling the false discovery rate (FDR). Here, FDR is calculated and then a selected threshold is applied. Otherwise, the number of false positives will be increased with the number of tests. Benjamini-Hochberg procedure (Benjamini and Hochberg, 1995) and permutation-based FDR estimates (Tusher et al., 2001) can be used to efficiently compute FDRs from *p*-value s to reduce the number of false positives. Such approaches can be performed using the p.adjust() function in R language (RStudio Team, 2020). In order to further remove background noise and identify the most significant differentially expressed proteins, fold-change can be used along with adjusted *p*-value cutoff. Fold change calculation is typically followed by log2 numerical transformation to center the distribution of the resulting values around 0. In all cases, follow-up studies are required to accurately describe biological phenomena underlying the detected changes (Dalman et al., 2012).

### Clustering Analyses

Identifying individual proteins differentially expressed between time points or conditions is often insufficient for extracting biologically relevant information from a proteomics experiment. Instead, grouping similar items (i.e., proteins or samples/conditions) may be necessary to enable the exploration of data patterns without getting lost in lists (Figure 2D). The biological basis for this is that proteins often act in groups and the expression of proteins participating in the same processes may be co-regulated (Do and Choi, 2008). Clustering can help with the necessary data grouping. Clustering is a machine learning technique used in pattern recognition, data mining, and bioinformatics. The goal of clustering is to distinguish similar from different. Specifically, similar items (e.g., proteins) are grouped into the same cluster based on a common parameter or parameters (e.g., relative fold expression change across time), while different items are found in distinct clusters.

There are two types of clustering: supervised and unsupervised. In supervised clustering, class labels are provided and are used to guide learning (see section “*K*-means Clustering”). In contrast, in unsupervised clustering, observations are not associated with class labels. Unsupervised algorithms are primarily used for pattern discovery. For example, they can be used for exploratory data analysis, where the aim is to generate hypotheses rather than verify them.

Clustering of temporal proteomic HPI data is primarily performed by unsupervised learning, due to the lack of information about known expression patterns at different time points. A fundamental weakness of unsupervised approaches is that they assume there is an underlying pattern within the data; therefore, outputs from such methods should be carefully statistically and experimentally validated (Do and Choi, 2008). Temporal quantitative proteomic HPI data can be most appropriately clustered based on protein expression differences (see section “Statistical Analysis of Quantitative Temporal Proteomics Data”). For instance, relative fold expression change could be included in the data matrix used for clustering (Figure 2D). Meunier et al. (2007) demonstrated the application of unsupervised hierarchical clustering for proteomic data mining and its potential for characterizing tumor samples. Likewise, clustering is frequently used in temporal proteomic studies to uncover temporal trends and molecular signatures for infectious disease (Olsen et al., 2006; Weekes et al., 2014; Yang et al., 2015; Hou et al., 2017; Lapek et al., 2017; Itzhak et al., 2019; Soday et al., 2019; Hashimoto et al., 2020).

Clustering of a matrix containing relative fold expression changes for N proteins across X time points can be achieved in one of three ways (Oyelade et al., 2016). One option is to cluster proteins with similar relative fold expression changes across X time points. Here, proteins are considered objects, while samples are regarded as features. The goal in this case is to identify groups of proteins with similar pattern of relative protein expression change across time. Such groups may indicate co-function or co-regulation (Thalamuthu et al., 2006). Another option is to cluster samples/time points across all proteins. In this case, samples are regarded as objects and proteins are regarded as features, and the goal may be to reveal, for example, the phenotypic structure of samples (e.g., cyclic changes with groups of samples from different time points exhibiting similar changes). The third option is to cluster the data matrix along both, protein and sample axes.

Grouping similar items into the same clusters and dissimilar items into different clusters requires ways to measure the (dis)similarity or distance between each pair of items. This is accomplished by means of distance (aka proximity, dissimilarity, or similarity) measures, which are at the core of distance-based clustering algorithms. The performance of the clustering algorithm depends on the efficiency of its distance measures, and the results may change depending on the distance measure (Shirkhorshidi et al., 2015) and the algorithm. The choice of the distance measure and the clustering algorithm depends on the dataset (e.g., if the data is log transformed). For example, Gibbons and Roth (2002) reported superior performance of Euclidean distance measure for ratio-based data and Pearson distance measure for non-ratio measurements. The most commonly used distance measures are: Minkowski, Euclidean, Manhattan, Cosine, Pearson correlation, and Spearman correlation distances. Their performance with high-dimensional datasets has been extensively reviewed elsewhere (D’haeseleer, 2005; Brusniak et al., 2008; Kerr et al., 2008; Shirkhorshidi et al., 2015).

Understanding clustering algorithms is a prerequisite for their proper application to the clustering of temporal HPI proteomics data. Clustering algorithms can be classified by a number of parameters (Oyelade et al., 2016). For example, Clustering algorithms can be categorized as exclusive (hard, or crisp) or overlapping (soft). Exclusive clustering assigns each input item (e.g., protein) to a single cluster, whereas overlapping (soft) clustering allows a data point to belong to more than one group (Kerr et al., 2008). In the remainder of this section, we discuss the principles of selected unsupervised clustering algorithms. We focus on algorithms frequently used in the analysis of temporal proteomic HPI data as well as on the algorithms we believe to be particularly useful for the analyses of such data.

#### Hierarchical Clustering

**FIGURE 3:**
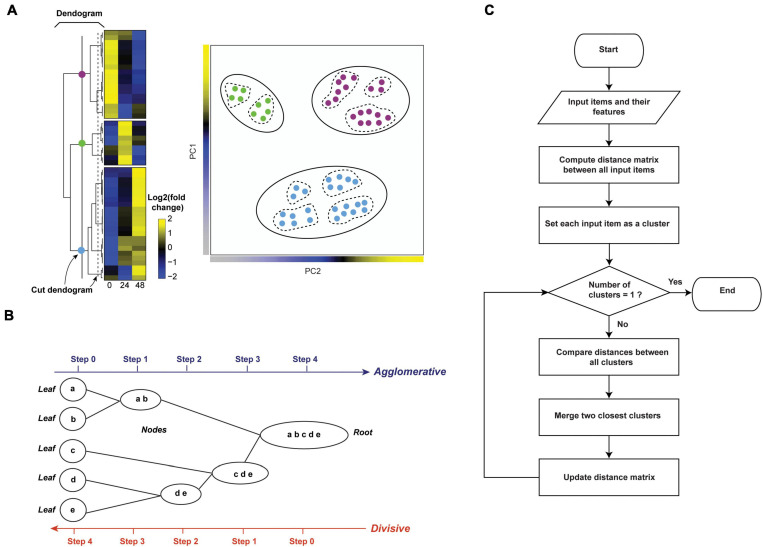
Hierarchical clustering. **(A)** Relative abundances of 50 proteins across three time points were clustered by hierarchical clustering (left panel). The dendrogram reflects relationships between proteins. The tree was cut at two levels indicated by colored circles and a dashed line to yield 3 and 9 clusters, respectively. The separation of subclusters is illustrated in the PCA plot in the right panel. Here, each dot represents a protein. **(B)** Agglomerative and divisive are the two types of hierarchical clustering. The agglomerative works in bottom-up manner, recording the sequence of cluster merges. The divisive algorithm works in a top-down manner, recording the sequence of cluster splits. **(C)** Flowchart of unsupervised hierarchical clustering algorithm (agglomerative).

In hierarchical clustering, all proteins are joined into clusters that form a nested dendrogram (aka a tree-shaped data structure, Figure 3A, right panel). The dendrogram reflects how similar or different objects (i.e., proteins and/or samples/time points) are across all features. The most similar objects are connected by clusters near the tree’s terminal branches (i.e., leaves. Figure 3B); root cluster connects objects that are most different. Therefore, in a hierarchical cluster, rows and/or columns, depending on the analysis option, are re-ordered placing similar objects close to each other. The data are transformed to color scale to help visualize the matrix (Figures 2D, 3A). The tree can be cut at varying levels to obtain the desired number of clusters (Figure 3A).

Hierarchical clustering algorithms work on distance measure matrices, which are calculated for each pair of objects using the input data matrix and the selected distance measure. Depending on how the clusters are formed, hierarchical clustering can be classified as agglomerative or divisive. Agglomerative clustering, also called agglomerative nesting (AGNES), or bottom-up clustering, works from the bottom up. It starts by treating individual objects as clusters, followed by computing distance measures between all pairs of clusters and then recursively joining the closest pairs according to their distance until a single cluster is made (Figures 3B,C). Here, intercluster distance, or “linkage function,” determines how distances between clusters are calculated, and which clusters are connected. The most common linkage functions are: minimum/single, maximum/complete, average/UPGMA (unweighted pair-group method using arithmetic averages), and centroid/UPGMC (unweighted pair-group method using centroids) (D’haeseleer, 2005). The distance between two clusters per minimum linkage function equals the distance between the closest two members of each of the two clusters; conversely, per maximum function it equals the distance between the furthest two members of each of the two clusters. Average and centroid functions calculate the distance between any two clusters as average distance between cluster members and as the distance between cluster centroids, respectively (D’haeseleer, 2005). Unlike agglomerative clustering, divisive clustering, aka divisive analysis (DIANA), is a top-down approach. It starts by including all objects in a single cluster and works to iteratively split the most heterogeneous cluster into components. This is repeated until all clusters are made up of single indivisible objects (i.e., proteins, Figure 3B; Karimpour-Fard et al., 2015). There are several methods for splitting clusters, see Roux (2018) for review. Agglomerative clustering is strongest at identifying small clusters and may deliver suboptimal performance in the detection of large clusters. Conversely, divisive clustering is best at identifying large clusters (Chipman and Tibshirani, 2006). To combine the strengths of the two approaches, Chipman and Tibshirani (2006) proposed a combined “mutual cluster” approach that informs the divisive approach by the results of an agglomerative analysis.

R has some useful built-in functions for performing hierarchical clustering. For instance, the hclust() function in stats R package (RStudio Team, 2020) and agens() function in cluster package (Maechler et al., 2019) are commonly used to perform agglomerative hierarchical clustering. Both functions include parameters that allow one to select the appropriate linkage and distance measures. Divisive clustering is often performed using diana() function in cluster package (Maechler et al., 2019). The number of generated clusters (Figure 3A) can be controlled by the cutree() function in stats package (RStudio Team, 2020). The mutual cluster approach is also available as mutualCluster() function in hybridHclust package in R (Chipman and Tibshirani, 2006).

The main weakness of hierarchical clustering lies in the dependence of results on various parameters, including distance measures and algorithm type. As a consequence, there is no one correct and true result (Oyelade et al., 2016). Therefore, the parameters of hierarchical clustering need to be tuned and the resulting clusters must be validated (see section “Evaluation Measures for Temporal Clustering”). Moreover, calculations can be computationally intensive, but previous steps (e.g., erroneous merging/division decisions) cannot be undone (Karimpour-Fard et al., 2015).

Despite its weaknesses, hierarchical clustering is a method of choice for visualizing and exploring large datasets, including temporal proteomic HPI data (Diamond et al., 2010; Weekes et al., 2014; Jean Beltran et al., 2016; Hou et al., 2017; Soday et al., 2019). For example, a study of Vaccinia Virus (VACV), the causative agent of smallpox, used multiplexed proteomics to quantify the changes of viral and host proteomes over a series of time points in infected vs. mock samples (Soday et al., 2019). Subsequently, agglomerative hierarchical clustering with centroid linkage and Pearson correlation distance measure revealed clear separation between infection stages, with the greatest changes occurring at late time points. Moreover, protein-level clustering identified multiple dysregulated pathways. This included, for example, the down-regulation of proteins involved in “cell attachment site” during infection, which suggested that VACV targets cell surface proteins to evade host’s defensive immune responses. Thereby, clustering helped make insights into biological processes modulated by VACV infection.

#### *K*-Means Clustering

**FIGURE 4:**
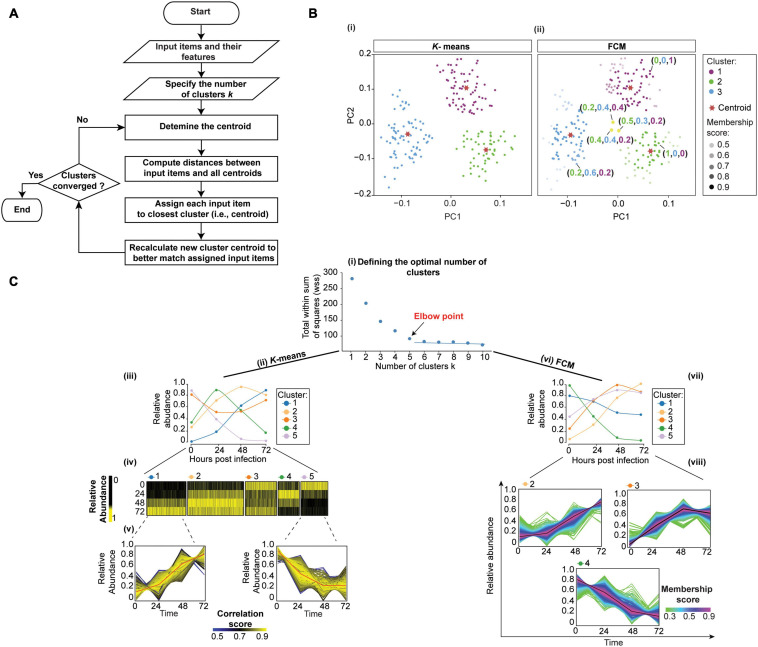
Clustering temporal HPI data using *K*-means (crisp clustering) and Fuzzy C-means (FCM, soft clustering) methods. **(A)** Flowchart of the unsupervised *K*-means algorithm. Initialization, iterations, and termination in FCM are the same as in the *K*-means algorithm. However, FCM uses a weighted centroid based on memberships of data points within each cluster because membership scores can vary from 0 to 1. **(B)** We illustrate the difference between *K-means* (i) and FCM (ii) by using mock temporal HPI data with 3-time points and principal components to display the data. (i) *K*-means divides data into three distinct clusters. (ii) On the other hand, FCM assigns each data point coefficients that reflect memberships in each of the clusters and range from 0 to 1. Then, each data point is assigned to the cluster in which its membership is highest. Some proteins (yellow dots) are assigned high membership coefficients to more than one cluster. **(C)** To demonstrate the application of *K*-means and FCM, we randomly selected 3,000 proteins from experiment 1 data by Weekes et al. (2014) and used the Elbow method to define 5 as the optimal number of clusters for both *K*-means and FCM; the plot for *K*-means is shown (i). We then used *K-*means (ii–v) and FCM (vi–viii) to cluster the data. The results of the *K*-means clustering are displayed (iii) as centroid (or average) temporal profiles of each cluster, and (iv) as heatmaps. (v) Each cluster can also be visualized by displaying the data for all proteins in the cluster, and coloring the corresponding protein profiles based on how well they correlate with the cluster’s centroid (red line); examples for clusters 1 and 5 are shown. Here, proteins with similar profiles to the cluster’s centroid have a score approaching 1 (yellow), while those with divergent patterns have a correlation score closer to 0 (blue). This plot can help filter proteins whose profiles don’t fit well into the selected cluster or identify core proteins of particular interest with profiles closely matching the centroid. Likewise, FCM results are displayed as the centroid temporal profile of each cluster (vii). (viii) Clusters can also be visualized by displaying each protein and color-coding its profile according to its membership in the respective cluster; plots for clusters 2, 3, and 4 are shown. Here, proteins with a profile close to the cluster’s centroid (black lines) have a membership score close to 1 (purple). These proteins are prominent members of a cluster (viii). In contrast, proteins with divergent patterns have a score closer to 0 (green). One may wish to apply a membership score threshold to eliminate proteins with divergent patterns from the downstream analyses of the cluster.

*K*-means clustering is an iterative algorithm that partitions the dataset into a predetermined *k* number of clusters, in a way that intra-cluster and inter-cluster similarities are maximized and minimized, respectively (D’haeseleer, 2005). The algorithm first shuffles the dataset and then randomly chooses *k* patterns as initial centroids for each cluster. After that, each data point (in our case, protein) is assigned to the cluster by finding the pattern’s closest centroid (a centroid is a data point at the center of a cluster) using proximity measures (e.g., Euclidean distance). The new centroid is then computed for each cluster by taking the average of all proteins assigned to that cluster. This process repeats until no more proteins change the cluster (Figures 4A,Bi).

To exemplify the application of *K*-means clustering to temporal HPI proteomics data, we randomly selected 3,000 host proteins from experiment 1 data by (Weekes et al., 2014). The first step in *K*-means clustering is to define the optimal number of clusters, which is critical for generating biologically meaningful groupings (Yang et al., 2015). For instance, the overestimation of parameter *k* will partition related proteins into different clusters, thereby confounding downstream inferences (Yang et al., 2015). The two most commonly used *k*-value selection algorithms are the Elbow method and the Average silhouette method (Yuan and Yang, 2019). The Elbow method, for instance, measures the variability within each cluster (i.e., within cluster-sum of a squares, WSS) as a function of the number of clusters (Figure 4Ci; Yuan and Yang, 2019). One should choose the number of clusters at but not after elbow point (Figure 4Ci). Here, we used fviz_nbclust() function in factoextra R package (Kassambara and Mundt, 2020) to define 5 as the optimal number of clusters (Figure 4Ci). We then applied kmeans() function in stats R package (RStudio Team, 2020) to cluster host proteins into five distinct clusters (Figure 4Cii). To observe the temporal profiles of each cluster, one may wish to visualize centroid profiles (Figure 4Ciii). Furthermore, temporal profiles of each group can be visualized using heatmap *via* heatmap() function in stats R package (RStudio Team, 2020) (Figure 4Civ). To identify core proteins whose expression closely matches the cluster’s centroid, one can correlate each protein in the cluster with the cluster centroid (Figure 4Cv).

*K-*means is one of the most straightforward clustering methods due to the ease of programming and computational efficiency. However, one of its drawbacks is that it generates hard and unrelated clusters. Therefore, it is not suitable for expression datasets containing overlapping clusters, or for assessing between-cluster relationships (Oyelade et al., 2016). Moreover, it works well in capturing the structure of the data if clusters have a spherical-like shape but performs poorly if clusters have complex geometric shapes. Therefore, it is not a good candidate for high-dimensional data and highly connected clusters (Oyelade et al., 2016). Furthermore, since *K*-means clustering is sensitive to outliers, it is always appropriate to remove the outliers before clustering. Additionally, *K*-means clustering is sensitive to initialization, meaning that the final clustering result depends on the position of the initial cluster centroid (i.e., seed). Therefore, it is essential to run the algorithm several times by applying different random seeds, or use an advanced version of *K*-means. For example, *K*-means++, available in pracma R package (Borchers, 2019), which runs a preliminary iterative algorithm to determine the most appropriate initial seeds.

*K*-means algorithm has been used in the analysis of temporal proteomic HPI data, rendering intuitively clear summaries of temporal patterns (Weekes et al., 2014; Clements et al., 2017; Hou et al., 2017; Lapek et al., 2017). For example, the aforementioned Soday et al. (2019) used *K-*means to cluster temporal profiles of co-expressed viral proteins. Subsequent functional enrichment of clusters revealed the co-expression of viral proteins involved in “Host interaction” and “DNA replication” at early time points, followed by virion-associated proteins at later times. Therefore, these temporal clusters revealed patterns in the expression of proteins with specific biological functions. Furthermore, the functions of uncharacterized pathogen proteins may be inferred *via* their associations with known proteins in their respective clusters, or by analyzing their expression in the context of changes occurring within the host. For instance, the authors compared viral temporal profiles with the inverted temporal profile of the host’s cellular protein HDAC5 using Euclidean distance. This matched C6 viral and HDAC5 host profiles, suggesting that C6 targets HDAC5 for proteasomal degradation. This was subsequently experimentally confirmed. Therefore, *K*-means clustering helped gain direct insights into viral-host interactions.

#### Fuzzy Clustering

Although hard clustering methods, including hierarchical and *K*-means, can accurately group distinct expression patterns, they are unable to identify input items (e.g., proteins) with similarities to multiple distinct clusters (Gasch and Eisen, 2002). For example, inaccurate clusters may be produced in the analysis of large datasets, where a protein has expression pattern similar to one group in one set of biological samples and another group for the remaining samples.

Several fuzzy clustering algorithms, including the fuzzy C-means clustering (FCM), have been developed to deal with such complicated relationships between objects (Bezdek et al., 1984, 1999; Friedman et al., 2000; Oyelade et al., 2016). FCM is very similar to *K*-means, and is likewise widely used. As in *K*-means, the number of clusters must be pre-determined. However, unlike *K*-means, FCM does not simply assign an input item (e.g., a protein) to a single cluster. Instead, FCM facilitates the identification of overlapping groups of data points by allowing each input item to belong to more than one cluster with a probability (membership coefficient) ranging from zero to one (Figure 4Bii). Proteins whose expression patterns are very similar to the center (or centroid) of a cluster will be assigned a high membership in that cluster (i.e., 1). Conversely, proteins that lie far away from the center of the cluster (i.e., with little similarity to the centroid) will have a low degree of membership to that cluster (i.e., 0). Therefore, FCM reveals the relative degree of each input item belonging to each of the clusters (Figures 4Bii,Cviii).

Like *K-*means clustering, FCM is sensitive to initialization and is affected by initial parameter values. One of these parameters is the *c*-value (Kerr et al., 2008; Oyelade et al., 2016), which defines the number of clusters and is equivalent to the *k*-value in *K*-means clustering. Approaches like the Elbow method are recommended to determine the optimal number of clusters. Another parameter is the fuzziness parameter *m* (Dembéle and Kastner, 2003). This parameter needs to be tuned, and one should apply validity indices to determine the optimal value of *m* (Zhou et al., 2014b). FCM is available as cmeans() in e1071 (Meyer et al., 2020), fanny() in cluster (Maechler et al., 2019), fcm() in ppclust (Cebeci, 2019), and mfuzz() in Mfuzz R packages. To demonstrate the application of FCM, we used mfuzz() function in Mfuzz R package (Kumar and Futschik, 2007) with the default value for parameter *m* to re-analyze data used for *K*-means clustering (Figure 4Cvi).

Despite limitations, FCM is frequently used particularly in phosphoproteomics studies to elucidate the dynamics of phosphorylation signaling events. Indeed, partitioning the identified phosphorylation sites into distinct clusters can help identify corresponding kinases and important regulatory events (Blagoev et al., 2004; Zhang et al., 2005; Olsen et al., 2006; Schmutz et al., 2013; Zhuang et al., 2013). For instance, Schmutz et al. (2013) used LC-MS/MS and FCM to study quantitative phosphorylation changes during *Shigella flexneri* infection. Most of the early phosphorylation events clustered together and were related to the regulation of cytoskeleton and cell adhesion.

#### Self-Organizing Map

Self-organizing map (SOM; aka Kohonen map, or self-organizing feature map, SOFM) is a an artificial neural network approach for reducing the dimensionality of input data in an organized manner that preserves the similarities between original input items (Kohonen, 1990). Since the output dimensionality is pre-defined for SOM, it is capable of reducing dimensionality further than PCA. Indeed, 2 or 3 components of PCA may not explain variability, but a SOM may provide a 2-dimensional rendering of relationships within the original data. Furthermore, like *K*-means clustering, SOM may not reproduce each item one-to-one from the original data on the output grid (i.e., mapping may be many-to-one). However, unlike *K*-means clustering, relationships between clusters are preserved. Therefore, SOMs allow for better visualization and interpretation of high-dimensional datasets than *K*-means, FCM, or PCA (Tamayo et al., 1999; Kohonen, 2014).

SOMs are particularly recommended for large and complex datasets, as simpler clustering approaches are available for small datasets (Kohonen, 2013). In other words, if simpler approaches do not produce satisfactory groupings for small datasets (e.g., hundreds of input items), SOMs should be applied. Conversely, with millions of input data items, clustering approaches described above may be less efficient than SOMs, and SOM analyses can be used at the outset. SOMs are useful, for instance, in medical image processing (Chang and Teng, 2007), astrophysics (Naim et al., 1997), industry (Simula et al., 1999), robotics (Sayers, 1991), and data mining. In biology and biochemistry (Tamayo et al., 1999; Olsen et al., 2006), it is frequently used for reducing data dimensionality: high-dimensional data is displayed in a reduced, usually two-dimensional space. In a SOM, similar objects are located close to each other and different objects are far apart, based on the characteristics of the input data. SOMs have been applied in proteomics (Schmidt et al., 2007; Peng et al., 2012) and temporal phosphoproteomics. For example, Zhang et al. (2005) identified modules of phosphorylation sites with similar temporal patterns within the epidermal growth factor receptor signaling network. Moreover, the potential functions of uncharacterized phosphorylation sites were inferred based on the functions of other components within the modules. For instance, hypothetical protein FLJ30532 was predicted to be involved in the immediate-early response to epidermal growth factor stimulation. Likewise, the algorithm can be applied to temporal proteomic HPI studies.

Various SOM variants are available. They all typically require users to specify the size and shape of the grid or array on which the output will be mapped. The size of the grid (with the corresponding number of nodes) needs to be determined by trial and error. If an output with fine details is expected, a larger grid should be used; however, if only coarse details are expected, a smaller grid will suffice. Indeed, too few output nodes (i.e., too few positions in the grid) can result in sizeable intra-cluster variation. In contrast, too many nodes can result in meaningless clusters (Kerr et al., 2008). A good starting point is 50 input data items per node (or location) on the grid (Kohonen, 2013). For example, a grid with 100 nodes (e.g., a 10 × 10 grid) would be used for five thousand input items. Various array shapes are possible, including a 2D rectangle or hexagon, or a 3D torus. From among 2D shapes, a hexagon is recommended for its visual clarity and accuracy (Kohonen, 2013). Oblong shapes are recommended due to faster convergence in learning with length and width corresponding to the two largest principal components (Kohonen, 2013). Depending on the data, a special shape of the output array may be justified. For example, relationships of data on the edges of a standard 2D rectangular output may not be represented effectively (Kohonen, 2013). Options exist to dynamically modify the shape and the size of the output array depending on the input data (Fritzke, 1994). However, in practice, border distortions should not pose issues for the use of SOM in proteomic data analysis or interpretation (Kohonen, 2014). Moreover, SOM algorithms rely on distance or similarity measures for determining how input items relate to each other. Euclidean distance is an example of a good measure that can be used for clustering proteomics HPI data (Giraudel and Lek, 2001).

**FIGURE 5:**
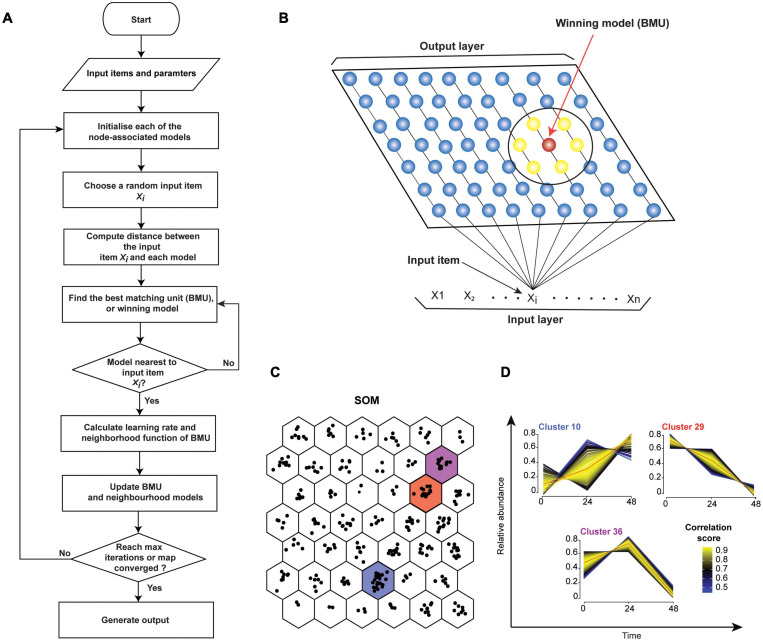
Self-Organizing Map (SOM). **(A)** Flowchart of a sequential SOM algorithm. **(B)** SOM input and output layers. Input items (x_1_ - x_*n*_) will be compared individually to models/nodes. The red model represents the best match, and the surrounding models (yellow, within the larger circle) are its neighbors. **(C)** Mock 3-time-point data was grouped into 42 clusters in a hexagonal 6 × 7 output grid using SOM. Points in each hexagon represent proteins. **(D)** Temporal profiles of proteins from panel (B) clusters 10, 29, and 36 are shown. Profile colors reflect the correlation of the protein profiles to cluster’s centroid (red line). This can help to select or filter proteins from clusters. Proteins with similar profiles to the cluster’s centroid have the correlation score approaching 1 (yellow), while those with divergent patterns have a correlation score closer to 0 (blue).

There are two main types of SOM algorithms. The first one is the sequential, or step-wise recursive SOM algorithm. The SOM using a step-wise recursive algorithm is constructed as follows (Figure 5A). One first selects a size and geometry of the output grid (e.g., a 2D 9 × 7 rectangle). A model is associated with each node of the grid. These models must be initialized before the algorithm is run (Kohonen, 2012). After SOM algorithm is completed, models essentially become the projections of the original data onto the output grid of the SOM. Random numbers can be used for initializing models. Alternatively, PCs (see PCA, in section “Quantitative Temporal Data Visualization, Preprocessing, and Quality Control”) can be used to initialize the models, thus speeding up the analysis. Here, PCs that describe the most variation are used. Subsequently, a random input item (x_*i*_, Figures 5A,B) is presented and compared to all the models. The distance between the input item and each model is calculated (e.g., using Euclidean distance) to find the best-matched model, or Best Matching Unit (BMU) (Figures 5A,B). The BMU’s neighborhood is then identified using a neighborhood function. Right after, BMU and its neighborhood models are updated to better match the input data item (x_*i*_). This is repeated for all input items (x_1_–x_*n*_) multiple times (Figure 5B). Neighborhood function and learning rate, which defines how adjustments are made to the model(s) at each step, are reduced with SOM algorithm iterations (see Kohonen, 2013). Hence, over time, the amount by which models are adjusted decreases. Therefore, SOM converges to show nonlinear projections of the input items (vectors) on the 2D output array (Figure 5C). Moreover, the correlation between each protein in the cluster and cluster’s centroid can be computed to identify core proteins whose expression closely matches the cluster’s centroid (Figure 5D). Several different options for defining learning rates and neighborhoods are available. A nonlinear learning rate and Gaussian neighborhood function typically produce robust results and are good starting points (Stefanovič and Kurasova, 2011).

The second main type of SOM algorithm is the batch algorithm. There are several benefits to using the batch SOM algorithms for the analyses of proteomic HPI data instead of the sequential SOM algorithm. Firstly, the learning rate parameter is eliminated. Therefore, the result is more robust and less affected by the user’s input. Secondly, it is faster than the step-wise method. When running a batch algorithm with Euclidean distance measure, it is recommended that the models be initialized by PCs. This speeds up the completion of the algorithm. In the beginning of the first training cycle of a batch SOM algorithm, all input data items are passed to each of the nodes in a grid. The input items matching each model at each node are saved in association with that corresponding node. Again, neighborhood function defines how and which nodes adjacent to the node with the best matching model will be modified. Then, adjusted model values are calculated for all nodes in all neighborhoods in one concurrent operation. The models are then updated, concluding one training cycle and brining values closer to the equilibrium. For datasets of up to a few thousand nodes, it is generally recommended that a training process incorporate the coarse and the fine training stages (Kohonen, 2014). Here, during the coarse stage a large neighborhood function equaling about 20% of the larger grid dimension is initially used. It should be reduced to about 5% of the smaller dimension over a few dozen training steps. During the fine training stage, the smallest neighborhood function that was reached during the coarse stage should be used. Each of the two training stages usually continues for several dozen steps until equilibrium is reached (Kohonen, 2014).

Some versions of SOMs combine the strengths of artificial neural networks (i.e., speed and robustness to noise) with other types of analyses to improve the overall performance of the algorithm (Dopazo and Carazo, 1997; Dogan et al., 2013). For example, the Self-Organizing Tree Algorithm (SOTA) integrates SOM and hierarchical clustering to quickly produce a dendrogram output while eliminating the need for pre-selecting the size of the output grid (Dopazo and Carazo, 1997; Yin et al., 2006; Kerr et al., 2008). The SOTA algorithm has a binary tree topology structure, in which the algorithm first selects a node with the largest heterogeneity and splits it into two nodes, called daughter cells. The tree’s growth continues until all observations (in our case proteins) map onto a unique leaf node (Kerr et al., 2008). The SOTA algorithm is implemented in the clValid R package (Brock et al., 2011). Another algorithm, called the adaptive double SOM (ADSOM) combines SOM and two-dimensional position vector analyses with parameter training (Ressom et al., 2003). This eliminates the biases arising from user-specified parameters while allowing quick and efficient clustering.

### Evaluation Measures for Temporal Clustering

The evaluation and validation of clustering are frequently excluded from analysis. However, they are essential for obtaining meaningful clusters (Bhargavi and Gowda, 2015). The quality of clusters can be evaluated computationally by external and/or internal measures. External measures evaluate the quality of clusters by comparing clustering-derived partitions to known labels, e.g., a gold standard dataset. Conversely, internal validation measures evaluate the quality of the clusters without any external data, by simply evaluating intra- and inter-cluster variation. The same internal methods are used to define, for example, the optimal number of clusters in *K*-means clustering (see section “K-means Clustering”). See Handl et al. (2005); Liu et al. (2010), Bruno and Fiori (2013), and Oyelade et al. (2016) for a comprehensive overview of internal and external measures and their biases.

Moreover, several biological measures are available for assessing the ability of clustering algorithms to generate biologically meaningful groupings. The most widely used biological measure is the functional enrichment analysis (Figure 2E). It typically assesses the overrepresentation of biologically meaningful categories within clusters, i.e., whether more members of a category belong to a cluster than expected by chance (Boyle et al., 2004). See Huang et al. (2009); Karimpour-Fard et al. (2015), and Chen et al. (2020) for an overview of relevant statistical and bioinformatics methods. Two additional biological measures are the biological homogeneity index, which evaluates the homogeneity of clusters, and the biological stability index, which captures cluster stability through clustering iterations with similar data sets (reviewed in Bruno and Fiori, 2013). The lists of biological categories and the assessment methods are determined by the study’s research question as well as the data.

Many temporal proteomics studies utilized functional enrichment analysis to evaluate the quality of clusters (Blagoev et al., 2004; Zhang et al., 2005; Diamond et al., 2010; Weekes et al., 2014; Jean Beltran et al., 2016; Hou et al., 2017; Lapek et al., 2017; Breen et al., 2018; Li et al., 2018; Soday et al., 2019; Itzhak et al., 2019; Hashimoto et al., 2020; Santana-Codina et al., 2020). Conversely, external measures are rarely considered. This could stem, in part, from the lack of suitable gold standard datasets. However, whenever possible, all three measures should be used to evaluate clustering results (Bruno and Fiori, 2013). There is a useful package in R called clValid (Brock et al., 2011) that includes many built-in functions for internal and biological measures. Moreover, this package contains built-in-functions for various clustering algorithms, including hierarchical, *K*-means, SOMs, and FCM. External validation can be performed by external_validation() function in the ClusterR package in R, which also includes a number of clustering algorithms (Mouselimis et al., 2020).

## Exploring Subcellular Proteome Organization During Infection

Localization of proteins within subcellular niches enables them to find their partners and substrates, and thus become functional. These subcellular niches include macromolecule assemblages, such as the ribosome or centrosome, as well as organelles, which are physically demarcated by a lipid bilayer (Christoforou et al., 2014). These organelles can change their number, localization, structure, and composition in response to an infection or an external stimulus (Jean Beltran et al., 2016). The corresponding compartmentalization of proteins is likewise highly dynamic, enabling a quick response (Borner, 2020). Indeed, controlled protein localization is crucial to cellular hemostasis and its aberrations are associated with disease (Kau et al., 2004; Luheshi et al., 2008; Laurila and Vihinen, 2009; Park et al., 2011; Valastyan and Lindquist, 2014; Beltran et al., 2017; Siljee et al., 2018).

**FIGURE 6:**
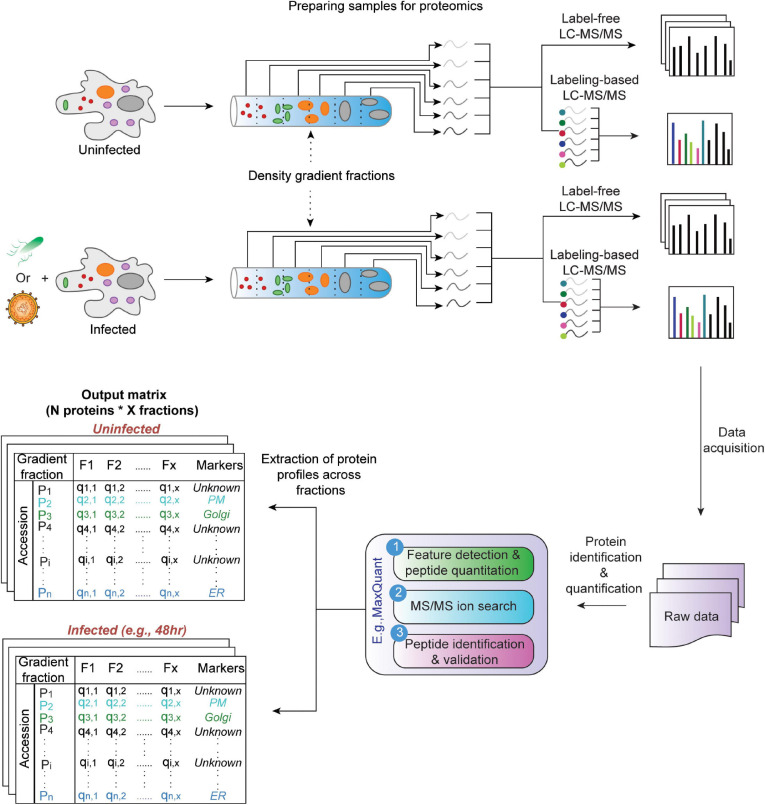
Schematic overview of gradient fractionation-based spatial proteomics. Infected and uninfected cells are lysed, and lysates are fractionated (Borner, 2020). The resulting fractions are then analyzed by label-free or multiplexed label-based quantitative MS. Proteins are then identified and quantified using specialized software (e.g., MaxQuant). This generates a data matrix containing relative protein abundances across fractions for *N* proteins along *X* fractions for infected and uninfected samples. Additional metadata (e.g., manually curated organelle markers) are added to the matrix to facilitate subsequent analyses. “Unknown” in the “marker” column indicates non-marker proteins with unknown localization.

Pathogen-induced localization alterations can occur throughout the course of an infection. These often include the placement of a pathogen’s proteins in the compartment as well as the reorganization of the host proteome. These changes must be mapped to understand disease progression. Here, we focus on the spatial proteomics (aka organelle proteomics) that uses fractionation and MS (Figure 6, reviewed by Gatto et al., 2010; Borner, 2020). Label-based localization of organelle proteins by isotope tagging (LOPIT) (Dreger, 2003) and label-free protein correlation profiling (PCP) (Foster et al., 2006) are two spatial proteomics approaches. They allow the detection of thousands of proteins in multiple subcellular compartments. Moreover, they improve the detection of low-abundance proteins compared to whole-cell proteomics. By thus increasing the depth of proteome coverage, they can help access specialized pathways manipulated by pathogens.

**FIGURE 7:**
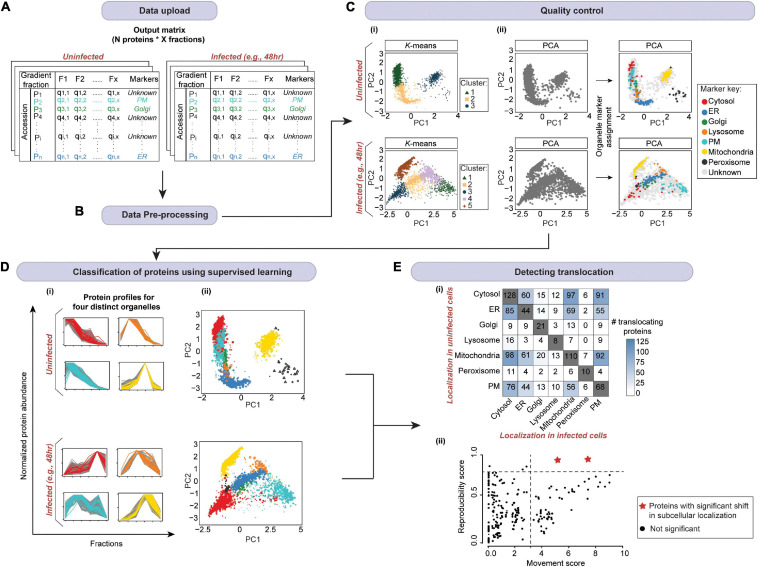
A schematic of spatial proteomic data analysis workflow for mapping localization changes in response to an infection. **(A)** After uploading the data (from Figure 6), **(B)** data pre-processing is carried out. This includes the imputation of missing values and normalization. Subsequently, **(C)** quality control is performed. (i) Here, first, unsupervised clustering can be applied to assess the overall data structure. (ii) Then organelle markers can be overlaid on the PCA plot to assess marker separation (ER, endoplasmic reticulum; PM, plasma membrane). **(D)** The organelle markers are then used to train a model (in supervised machine learning). The model will assign the profiles of proteins with unknown localization to organelles based on their similarity to the profiles of markers. Here, we randomly selected 3,000 proteins from Jean Beltran et al. (2016) uninfected and 48 h post-infection samples, and used SVM in pRoloc package in R (Gatto et al., 2014b) to assign protein localizations. (i) Profiles for four organelles from each condition are shown; marker key is same as in panel **(C)**. The plots demonstrate that non-marker and marker proteins that were predicted to colocalize by SVM exhibit similar profiles across the gradient. (ii) The whole multivariate data can then be visualized in two dimensions using PCA or t-SNE to portray organelle separation. Here, all proteins with localizations predicted by SVM as well as markers are colored according to the organelle they are assigned to; marker key is same as in panel **(C)**. **(E)** Finally, localizations in infected and uninfected cells are compared to determine (i) the most affected organelles, and (ii) candidate proteins with significantly shifted subcellular localization (red stars) by computing movement and reproducibility scores for each protein. Note, data from **(D)** was used for panel (**E**i); mock data was used for panel (**E**ii).

The output of both of these approaches are relative protein abundances across fractionations. These can be displayed, for example, as a matrix (Figures 6, 7A). Subsequently, pattern recognition is used to assign proteins to specific sub-cellular compartments by relating their localizations to known organelle markers (Gatto et al., 2010). At the same time, actual residents of organelles are distinguished from contaminants without the need for high-purity organelle isolations (Gatto et al., 2014a). Below, we present a robust workflow for spatial proteomics data analysis. It is implemented in pRoloc Bioconductor package (Gatto et al., 2014b) in R. Moreover, we update the existing workflows by presenting additional statistical approaches and algorithms, along with their principles, pros, and cons. In addition, we illustrate how these steps work by applying them to *in silico*-generated or published HPI datasets.

### Preparatory Steps

#### Organelle Markers

The prediction of protein localization in spatial proteomics traditionally depends on supervised machine learning (see section “Predicting Protein Localizations in Each Condition”), wherein a list of “bona fide” organelle markers (i.e., proteins with known localizations) retrieved from public databases (i.e., labeled training dataset) is utilized to map proteins of unknown localization to subcellular compartments. See Christoforou et al. (2014); Gatto et al. (2014a), and Borner (2020) for a detailed explanation on organelle markers and how to curate a set of reliable markers. Mapping spatial protein dynamics during an infection represents a special challenge as marker localizations may change. Therefore, it is essential to carefully select and validate markers for spatial proteomic HPI studies (Beltran et al., 2017).

**FIGURE 8:**
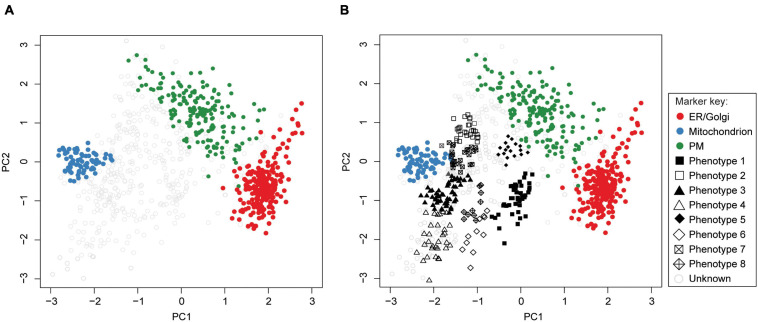
PhenoDisco algorithm helps assign localizations to proteins. First, the algorithm uses a minimal set of markers and unlabeled data as input to detect new phenotypes (i.e., clusters) *via* unsupervised learning. These are then used in supervised learning to predict protein localizations. **(A)** *Drosophila melanogaster* data (Tan et al., 2009) and ER/Golgi, Mitochondrion, and PM markers were used as input (ER, endoplasmic reticulum; PM, plasma membrane). **(B)** The phenotypes we identified by using phenoDisco correspond to ribosomal subunits (phenotypes 1 and 3), proteasome (phenotype 2), nucleus (phenotype 4), peroxisome (phenotype 6), and proteins with uncertain localization (phenotypes 5, 7, 8).

Supervised methods, such as support vector machines (SVM) have been used to map proteins to organelles with good accuracy (Trotter et al., 2010). The application of such methods, however, is limited by the availability of the organelle marker training datasets. Specifically, all localizations existing in the experimental output must be represented in the training dataset. If this condition is not met, protein localizations may be predicted incorrectly (Breckels et al., 2013; Christoforou et al., 2014; Gatto et al., 2014a). To address this, Breckels et al. (2013) developed phenoDisco—a semi-supervised machine learning schema to identify putative subcellular phenotype groupings in spatial proteomics experiments (Figure 8). The phenoDisco algorithm first uses novelty detection (unsupervised learning) to cluster the training data and expose the representative labels of each cluster/phenotype. Secondly, the algorithm examines and extracts points closest to cluster centroid for use as labeled training instances (Christoforou et al., 2014). These labeled training instances need to be carefully validated before the subsequent round of supervised learning that will use them alongside markers to assign protein localizations. The phenoDisco algorithm is available in pRoloc R Bioconductor package (Gatto et al., 2014b). The ability to identify and examine clusters offered by this algorithm is particularly useful in the study of infectious disease, which might trigger protein localization changes.

#### Dimensionality Reduction Tools for Visualizing Organellar Map

**FIGURE 9:**
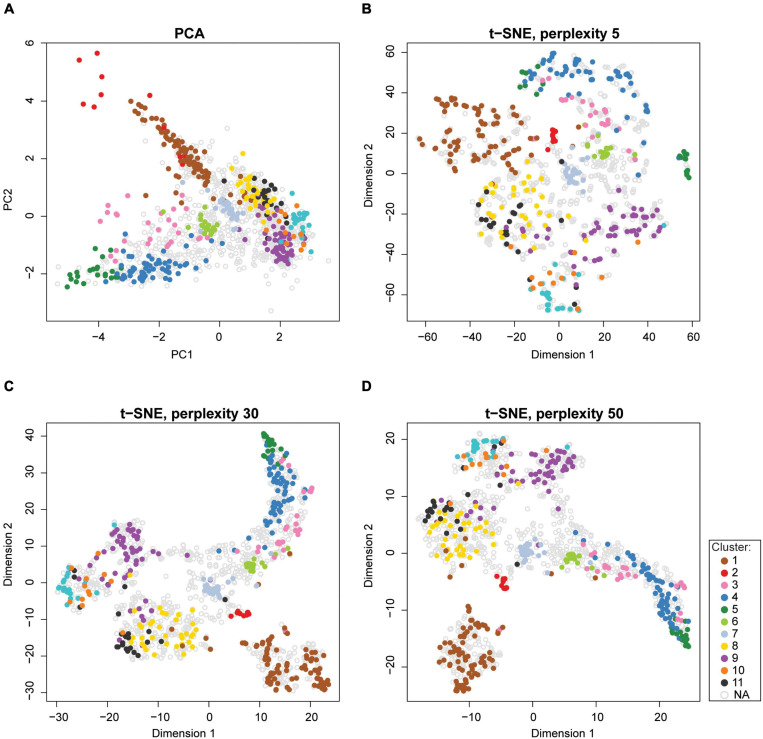
Visualization of high-dimensional spatial proteomics data using PCA and t-SNE. **(A)** Data can be displayed along principal component values (PCs) of PCA in two or more dimensions. **(B–D)** Better resolution of clusters can be achieved by t-SNE. However, the results of the t-SNE algorithm depend on the values of its tunable parameters. For instance, the perplexity parameter typically ranges between 5 and 50 (Van Der Maaten and Hinton, 2008). Perplexity settings of 5, 30, and 50 are shown in panels **(B–D)**. All other parameters were kept constant. Human Embryonic Kidney (HEK293T) sample data from pRoloc package was used for all plots.

Dimensionality reduction techniques are a convenient tool for visualizing high-dimensional spatial proteomics data (Figure 9). There are two types of dimensionality reduction techniques: linear transformation (e.g., PCA, refer to section “Quantitative Temporal Data Visualization, Preprocessing, and Quality Control” for more details on PCA) and non-linear transformation (e.g., t-SNE, t-distributed stochastic neighborhood embedding, or UMAP, uniform manifold approximation and projection). t-SNE finds a pattern in the data by calculating pairwise similarities between points in the high-dimensional space, and projects this onto a low-dimensional space, progressively minimizing the difference between the two sets of similarities while preserving the local structure of the data (Van Der Maaten and Hinton, 2008). Due to the probability distribution used to measure the embedding, t-SNE produces better-resolved clusters in a map, which makes it popular for visualizing subcellular clusters (Jean Beltran et al., 2016; Orre et al., 2019). Unlike PCA, t-SNE does not work on global variance and instances that contribute the most to the variability. Instead, t-SNE groups similar input items and emphasizes the separation of different input data items; thereby, the neighboring points in the high-dimensional space end up close to each other in low-dimensional space (Van Der Maaten and Hinton, 2008). However, the t-SNE algorithm is sensitive to its tunable parameters (i.e., perplexity, learning rate, and maximum iterations). Indeed, even slightly different parameter values can generate different output, making such maps difficult to compare (Figures 9B–D). Therefore, tunable parameters must be optimized. Furthermore, higher iterations of t-SNE result in better quality maps at the expense of longer runs; while too few iterations may not resolve clusters (McInnes et al., 2018). The best practice involves optimizing parameters using a grid-search (i.e., testing all combinations of parameters, refer to section “Predicting Protein Localizations in Each Condition”), and choosing the best combination for a given dataset (Orre et al., 2019). t-SNE algorithm is implemented in the tsne package (Donaldson, 2016) in R, as well as the pRoloc Bioconductor package (Gatto et al., 2014b).

Unlike t-SNE, which is a locally focused method, the UMAP preserves both local and global structure. It also boasts shorter run times and applicability to big datasets (Allaoui et al., 2020). Briefly, UMAP uses the *k-*nearest neighbor concept and builds a high-dimensional graph of the input data. It then optimizes the layout of the low-dimensional output graph to make it as similar as possible to the high-dimensional graph of the input data. The constructed high-dimensional graph is weighted, with edge weights showing the possibility that two points are connected. The UMAP algorithm extends the radius around each point and connects each point and its neighbors with intersecting radii to determine connectedness. The choice of the radius is essential, as isolated clusters can result from radii that are too small, and vice versa. However, UMPA deals with this issue by restricting the size of the local neighborhood when learning the manifold structure of data. Once the high-dimensional graph is constructed, it optimizes the graph’s low-dimensional embedding through stochastic gradient descent (Sainburg et al., 2020). Like t-SNE, the UMAP algorithm is sensitive to its tunable parameters (i.e., n_neighbors, the number of nearest neighbors; min_dist, minimum distance between two points), which can affect the balance between the local and global structure in the final projection. Similar to t-SNE, the tunable parameters must be optimized by using grid-search. The UMAP algorithm is implemented in the umap package (Konopka, 2020) in R. Since UMAP preserves both, the local and the global data structure, and is faster than t-SNE, it is typically preferred in data dimensionality reduction applications.

Note that the aforementioned techniques are mainly used to visualize the overall structure of the data and should not be used for assigning localizations to proteins. In particular, they can be used to evaluate marker proteins, to assess the resolution, tightness, and similarity of clusters, and to inspect whether the data has a well-defined structure (Figure 7Cii).

#### Data Preprocessing and Quality Control

Proteins identified by spatial proteomics are first annotated based on their localization as markers or non-marker proteins (Figure 7A). Marker proteins will subsequently be used as reference points to find new proteins with the same localization pattern (Gatto et al., 2014a). The next step in the analysis is to evaluate the quality of the dataset and collect descriptive statistics as detailed for temporal data in section “Quantitative Temporal Data Visualization, Preprocessing, and Quality Control”. Similarly to temporal proteomic data analysis, missing values must be imputed, and data normalized to generate reliable and comparable results for downstream analyses (Figure 7B).

##### Missing Value Imputation

A number of algorithms used for spatial proteomics data analysis cannot deal with incomplete data. However, the impact of imputation has not been thoroughly addressed in spatial proteomics literature. The raw quantitative data may contain missing ion intensity values for a number of reasons. These include low protein abundance and low instrument sensitivity (Karpievitch et al., 2012). For low abundance proteins, missing values can be imputed by substituting lowest observed intensity or peptide count (Karpievitch et al., 2012). This is computationally easy and fast. However, this does not take into account the patterns in the data and may introduce bias in the data as the number of imputed values increases. Another approach is to exclude proteins with missing values (Dunkley et al., 2006; Du et al., 2008; Hall et al., 2009; Tan et al., 2009). This is a suitable approach if only a small number of such proteins were identified; however, if many proteins are missing data, simply dropping them may increase the bias.

The effect of imputation on the downstream analysis of microarray data has been studied (see Oh et al., 2011 for an overview of different imputation methods). The same approaches are used in spatial proteomics. For instance, the *k*-nearest neighbors (*k*-NN) approach uses feature similarity to assign a value to a random missing point. It finds proteins (i.e., *k*-NN) with expression profiles similar to that of the protein with the missing data. A weighted average based on *k*-NN is then used to impute the missing value. Although this approach is accurate, it is sensitive to outliers (Kim et al., 2005). Another useful approach for imputing missing values is the Multivariate Imputation by Chained Equation (MICE) (Van Buuren and Groothuis-Oudshoorn, 2010). It assumes that the data are missing at random, and calculates multiple imputations with their corresponding estimates of uncertainty. Both, *k*-NN and MICE are suitable for datasets with a several data points missing at random. They are implemented in bnstruct (Franzin et al., 2017) and mice (Van Buuren and Groothuis-Oudshoorn, 2010) R packages, respectively. The *k-*NN approach is also available as impute() function in the DEP Bioconductor package (Zhang et al., 2018).

In cases when many data points are missing not at random, for example when proteins are not quantified in a specific condition (i.e., are below the detection limit), simply excluding such data from analyses will introduce bias (Luo et al., 2009). The missing values in such data can be imputed for example by using quantile regression-based left-censored function (“QRILC”). This approach is available as impute() function in the DEP Bioconductor package (Zhang et al., 2018). Values missing due to random chance (e.g., technical variability) and missing not at random can also be imputed by MSstats Bioconductor package in R (Choi et al., 2014).

However, all aforementioned imputation approaches are likely introduce bias (Karpievitch et al., 2012; Wei et al., 2018; Goeminne, 2019). For example, Gatto et al. (2014a) imputed missing values in a spatial proteomics dataset using *k*-NN, and the results generated from the imputed data indicated the misclassification of protein localizations. The best way to obtain unbiased estimates for missing values is to explicitly model the missing data by using methods, such as maximum-likelihood model (Karpievitch Y. et al., 2009; Kang, 2013), Bayesian (Luo et al., 2009), and Expectation-Maximization (EM) approach (Kang, 2013). These modeling approaches are applicable to data missing at random and not at random. Irrespective of the imputation method, the effect of data imputation must be carefully evaluated each time.

##### Data Normalization

Another critical aspect of data preprocessing is data normalization. Normalization methods must make samples statistically comparable, while correcting for intragroup differences (e.g., batch effects) and preserving between-group differences (e.g., differences between organelles) (Välikangas et al., 2018; Chen et al., 2020). The most common normalization approach for reducing unwanted variation in spatial proteomics involves dividing each ion intensity (or spectral counts) by the sum or maximum of ion intensities (or spectra counts) in each row (Gatto et al., 2014a; Breckels et al., 2016) or column (Hu et al., 2019). Such normalization can be done quickly and easily (Gatto et al., 2014a; Breckels et al., 2016; Hu et al., 2019). Callister et al. compared linear regression, central tendency, locally weighted regression, and quantile normalization methods (Callister et al., 2006). They all decreased systematic bias in the data, but linear regression normalization performed best. Linear regression assumes that bias and peptide abundance are linearly dependent. This means that as the measured peptide ion abundance increases, the systematic bias also increases (Callister et al., 2006). Rlr, RlrMA, and RlrMA (Välikangas et al., 2018) are linear regression variants available in MASS (Venables and Ripley, 2002) R package.

Similarly, Välikangas et al. (2018) systematically evaluated 11 popular normalization methods using four proteomic datasets. Results indicated that variance stabilization normalization (Vsn) reduced the intragroup variation the most and performed well in differential protein expression analysis with all tested datasets. Likewise, local regression normalization and linear regression normalization performed well. Moreover, excellent performance of Vsn was demonstrated by Gatto et al. (2014a), who used two biological spatial proteomic replicates with substantial technical variability to simulate multiple conditions. Vsn significantly reduced variation and improved overlap between replicates. Vsn aims to remove the dependencies between sample variances and mean intensities, and scale the samples to the same intensity range by using maximum likelihood estimation and parametric transformation (Huber et al., 2002). This statistical method is available as justvsn() function in the Vsn Bioconductor package (Huber et al., 2002) and normalize_vsn() function in the DEP Bioconductor package (Zhang et al., 2018). As in temporal proteomics experiments, the efficiency of normalization must be evaluated, e.g., using MA plots (see section “Quantitative Temporal Data Visualization, Preprocessing, and Quality Control”).

Several other normalization methods are available. Among these are LOWESS (locally weighted scatterplot smoothing) regression, and EigenMS (Quackenbush, 2002; Bolstad et al., 2003; Callister et al., 2006; Leek and Storey, 2007; Karpievitch Y.V. et al., 2009; Zhang et al., 2015). EigenMS uses singular value decomposition (SVD) on model residuals to capture trends that lead to the formation of bias. This algorithm is capable of handling missing values and is available as a stand-alone function implemented in R at http://sourceforge.net/projects/eigenms/ (Karpievitch Y.V. et al., 2009; Karpievitch et al., 2014).

After data preprocessing, the best practice is to use unsupervised clustering to assess the overall structure of the data (see section “Clustering Analyses”; Figure 7Ci). According to the De Duve’s principle (De Duve and Beaufay, 1981), proteins belonging to the same organelle will co-fractionate, thereby leading to the formation of distinct groups upon clustering. The whole dataset can also be visualized using dimensionality reduction techniques, such as PCA or t-distributed stochastic neighbor embedding (tSNE) without the addition of organelle markers to the map (Figure 7Cii; *left panel*). Indeed, overlaying markers at this stage can confer a false sense of structure to the data, precluding the visualization of the data’s overall structure (Gatto et al., 2014a). The lack of structure in the data is indicative of the clustering algorithm’s inability to separate clusters at later analysis stages. Next, organelle markers are overlaid (on plots or clusters) to assess the resolution in the data (Figure 7Cii; *right panel*). Here, a clear separation of markers by organelle membership is expected. Its absence will undermine all subsequent analyses and interpretation. Such a situation may be remedied by adjusting imputation and normalization methods. Furthermore, these overlaid maps can also be inspected for outliers, for instance an unexpected marker position. These may indicate unreliable protein quantitation, identification, or annotation as a marker (Gatto et al., 2014a).

### Predicting Protein Localizations in Each Condition

**FIGURE 10:**
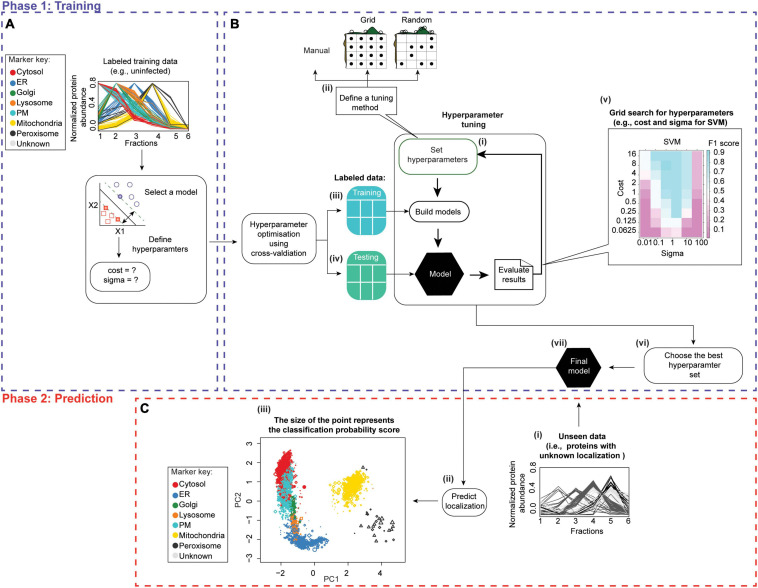
Schematic overview of supervised learning for subcellular localization mapping. **(A)** The first step in multiclass classification is to select an algorithm with its corresponding hyperparameters. Here, we show SVM. **(B)** Next, the hyperparameters must be tuned. This includes: (i) defining the range of possible hyperparameter values, (ii) selecting a method for sampling hyperparameter values (i.e., manual, grid, or randomized), and choosing a cross-validation technique (e.g., *k*-fold) for evaluating the model’s performance with labeled data correspondingly divided into training (iii) and testing (iv) datasets. Moreover, (v) a metric to judge the model’s performance with each set of hyperparameters must be defined. Based on the metric, the best set of hyperparameter values is chosen (vi). For instance, (v) the two SVM hyperparameters (i.e., cost and sigma) were optimized over 50 rounds of 5-fold cross-validation through a grid search, and then the best pair of hyperparameters was chosen based on the evaluation of *F1* score. Once the best combination of hyperparameters has been selected (vi), it can be used to build the final model (vii). **(C)** Proteins with unknown localizations are then presented to the model (i), and the model predicts their labels (i.e., localizations; ii). (iii) Classification scores for unlabeled instances that correspond to their most likely subcellular compartment are reflected by point sizes. Each point here represents a protein. Uninfected data from Figure 7 was used throughout this figure. The same workflow can also be applied to the infected data.

Supervised machine learning is the method of choice for predicting the subcellular localizations of proteins (Figures 7D, 10). During training, a supervised learning algorithm will learn to associate independent variables (i.e., protein abundances across fractions) and protein labels (i.e., marker assignments). After training, the algorithm predicts the labels for proteins with unknown localizations (Swan et al., 2013). Supervised learning algorithms can be subdivided into two groups based on the characteristics of the label: classification (labels are discrete categories) and regression (labels are continuous numeric values). In spatial proteomics, multiclass classification algorithms are typically used: labels are discrete and cover many (i.e., three or more) possible localizations. The first step in multiclass classification is selecting the algorithm (Figure 10A). Among common multiclass classification algorithms are naïve Bayes, *k*-nearest neighbor, SVM, random forest, and artificial neural networks. See Kotsiantis et al. (2007) and Crisci et al. (2012) for a comprehensive overview of supervised machine learning algorithms and their biases. However, it is widely accepted that how the algorithm is applied and the quality of the training data have a greater effect on the final result than the choice of the algorithm (Gatto et al., 2014a).

The second step in multiclass classification is defining the range of possible hyperparameter values (Figure 10Bi). Hyperparameters are adjustable parameters that have to be set before training to obtain a model with optimal performance. A model is defined by the combination of the selected classification algorithm and a specific set of hyperparameters. This model is then used to predict class labels. Examples of hyperparameters are: the regulation and constant parameter C in SVM algorithm, the number of nearest neighbors used (*k*) in *k*-nearest neighbors, and the number of decision trees in random forest. See Luo (2016) for a comprehensive overview of hyperparameters for different machine learning algorithms. Using default hyperparameter settings cannot ensure optimal learning performance. Moreover, wrongly selected parameters can adversely impact the resulting model’s performance (Gatto et al., 2014a; Luo, 2016; Probst et al., 2019; Schratz et al., 2019). Therefore, various combinations of hyperparameters must be tested to choose the best set.

Defining which hyperparameter combinations will be evaluated is the third step in multiclass classification (Figure 10Bii). Manual selection can be inefficient. Random or grid search allow for automated and efficient selection of hyperparameter combinations. In grid search (also known as exhaustive search), every possible combination of parameters within a specified grid is selected for subsequent evaluation (Rojas-Domínguez et al., 2017). In a random search, a fixed number of random combinations of hyperparameters is selected (Bergstra and Bengio, 2012).

Next, i.e., fourth step is the testing of all selected hyperparameter combinations by means of cross-validation. Cross-validation (out-of-sample testing) is a model evaluation method that estimates how accurately the model will predict the labels of unseen (i.e., out-of-sample) data (Payam et al., 2009; Figures 10Biii,iv). During cross-validation, labeled data is split into a training set (to train the classifier; Figure 10Biii) and a testing set (Figure 10Biv), which is used to evaluate the model’s performance with each pre-selected combination of hyperparameters. Subsequently, different evaluation metrics (see below) are used to evaluate the model’s performance (Figure 10Bv), and the hyperparameter combination resulting in the best model performance is selected (Figure 10Bvi).

Among the most common cross-validation techniques are the: (i) holdout, (ii) *k*-fold, (iii) leave-one-out, and (iv) leave-p-out methods. See Payam et al. (2009) for an overview of cross-validation techniques and their biases. For example, *k*-fold cross-validation has been used frequently in proteomic studies (Granholm et al., 2012; Swan et al., 2013; Hu et al., 2019). In *k*-fold cross-validation, the data is randomly split into *k* number of equally sized folds (i.e., groups). Then *k* iterations of training and testing are performed with a single combination of hyperparameters, such that at each iteration a different *k* fold is held-out as the test data set to evaluate the model’s performance, and the remaining (*k -* 1) folds are put together to form a training set. After each iteration, testing accuracy metric (see below) is computed using the testing dataset (i.e., the left-out fold). The overall out-of-sample accuracy is the average of all *k* iterations performed with a given set of hyperparameters (Payam et al., 2009).

During cross-validation, a confusion matrix is typically used to gain insight into the model’s performance and errors (Kautz et al., 2017). A confusion matrix is an N^2^ matrix (N is the number of classes) that compares the number of actual assignments to N classes with the number predicted by the model (Table 2). Based on whether the classes were correctly predicted by the model, observations can be categorized as true positive (TP, correctly identified), false positive (FP, incorrectly identified), true negative (TN, correctly rejected), and false negative (FN, incorrectly rejected). Then, based on the confusion matrix-derived categorization, performance metrics (Table 3) can be calculated and used to assess how well a given model performs on a testing data set (Figure 10Bv). The fifth step in multiclass classification is to choose the best combination of hyperparameters that maximize the model’s performance (Figure 10Bvi) and use that combination to build the final model from the training data (Figure 10Bvii). The sixth step is predicting class labels for unseen testing data (Figures 10Ci,ii). Here, proteins with unknown localizations are assigned classification scores that reflect their most likely localization (Figure 10Ciii). The seventh step is evaluating model performance with the testing dataset. This can be done for example, by performing functional enrichment analyses (see section “Evaluation Measures for Temporal Clustering”).

**TABLE 2:** A confusion matrix with *N* = 2.

	Predicted: No	Predicted: Yes
Actual: No	TN	FP
Actual: Yes	FN	TP

**TABLE 3:** Performance metrics.

Performance metrics	Definition	Formula
Accuracy	The ratio of the number of correctly predicted observations to total observations	$\frac{TP+TN}{TP+FP+FN+TN}$
Sensitivity or recall	The proportion of positives that are correctly identified as positive by the model	$\frac{TP}{\left(FN+TP\right)}$
Specificity	The proportion of negatives that are correctly identified as negative by the model	$\frac{TN}{\left(TN+FP\right)}$
Precision	The proportion of true positives out of all predicted positives	$\frac{TP}{\left(TP+FP\right)}$
F1 Score	The harmonic mean of precision and recall	$2\frac{precision\times recall}{precision+recall}$

All classification algorithms mentioned in this section have been implemented in the pRoloc package (Gatto et al., 2014b). Moreover, the caret R package (Kuhn et al., 2020) has 233 built-in classification algorithms and several functions for cross-validation and hyperparameter tuning using grid and random search. To demonstrate the application of supervised learning, we took the data from infected (i.e., 48 h post infection) and uninfected samples from Jean Beltran et al. (2016). We then randomly selected 3,000 host proteins and used the aforementioned framework to predict subcellular localizations (Figures 7D, 10). First, the labeled training data were constructed by mapping the markers available in the pRoloc package to the selected 3,000 proteins (Figure 10A). We chose to use the SVM implemented in the pRoloc Bioconductor package (Gatto et al., 2014b) to assign protein localizations (Figure 10A). Two hyperparameter values of the SVM model, cost and sigma, were optimized over 50 rounds of 5-fold cross-validation through a grid search (Figures 10Bi,ii,iii,iv). *F1* score metric was then used to evaluate the model’s performance and select the best parameters that result in the highest out-of-sample testing accuracy (Figure 10Bv). The optimized model was then utilized to predict the label for each protein with unknown localization (Figures 10Bvii,C).

### Detecting Protein Translocation Events

One of the main applications of spatial proteomics in the context of infectious disease is comparing organellar proteome maps (e.g., infected vs. uninfected) to identify proteins with altered subcellular localization (Jean Beltran et al., 2016). A simple contingency table tracking the total number of assignments to each of the compartments in each map can be used to assess the global pattern of change (Figure 7Ei). However, this method is prone to error. For instance, situations when identities of proteins in each compartment change but their numbers remain would be missed (Borner, 2020).

A more sensitive assessment of localization changes evaluates each protein individually (Figure 7Eii; Gatto et al., 2014a; Itzhak et al., 2016; Borner, 2020). Indeed, if a protein’s localization changes during an infection, it’s profile, or abundance/enrichment across compartments would change as well. Conversely, if a protein’s localization were not affected, its profile would remain the same in infected and uninfected samples. Moreover, the profile’s change (or lack thereof) should be reproducible across replicates. This is the basis for detecting protein localization changes by means of MR plots (Figure 7Eii). Here, M stands for the magnitude of translocation score, which compares the profiles between two conditions, and R stands for the reproducibility of translocation score. See Itzhak et al. (2016) for details and formulas. Note that replicates are essential for the statistical power of this test, and cut-offs are determined based on the comparison of two sets of control data (e.g., uninfected) samples. In an MR plot, significantly translocating proteins are found in an upper right-hand quadrant (Figure 7Eii; red stars).

However, when infection results in drastic morphological alterations, and significant changes in profiles of most proteins are observed, it might be better to identify translocation events based on altered predicted compartment association (Jean Beltran et al., 2016).

## Discussion

Advances in sample preparation methods, mass spectrometry, as well as computational facilities and approaches allow producing and analyzing a plethora of proteomic HPI data to reveal changes occurring across space and time in response to an infection. Analyses of spatial and temporal proteomic HPI data can be especially challenging due to the data’s high complexity. In this review, we present the workflow pipelines for the analysis of such datasets. Moreover, we discuss the pros, cons, best practices, and challenges associated with each step. Novices in the field can use this review as a workflow tutorial, while experienced users may find it helpful for updating their data analysis pipelines.

Numerous pathogenesis strategies exist. Since only a small portion of spatial or temporal infection-related proteome changes has been mapped, and only for a small subset of pathogens (Sánchez-Quiles et al., 2011; Gudleski-O’Regan et al., 2012; Weekes et al., 2014; Matheson et al., 2015; Greenwood et al., 2016; Jean Beltran et al., 2016; Karniely et al., 2016; Soday et al., 2019; Depierreux et al., 2020; Tiku et al., 2020), we expect to see many more publications in the area of spatial and temporal HPI proteomics. Moreover, since disease progression is a dynamic process, simultaneous analyses of multiple subcellular compartments at critical time points throughout the course of an infection (i.e., a combined spatiotemporal study) can help understand disease processes on the molecular level better than each of the approaches alone. An example of such a study is the analysis of organelle alterations occurring during the course of human cytomegalovirus infection (Jean Beltran et al., 2016). Here, essentially, spatial proteomics experiments were repeated across a series of time points post-infection. The resulting maps defined protein trafficking in space and time, and elucidated essential disease processes and protein roles.

The next level of complexity in investigating HPIs is the mapping of protein complexes and protein-protein interactions on a global scale with resolution in space and time. This is a critical component of understanding how the observed changes are orchestrated. Future experimental and computational efforts will be moving in this direction. Moreover, bioinformatics pipelines will be developing to better integrate spatial and temporal maps of proteome and protein complex changes during disease progression. Furthermore, since pathogens alter multiple interconnected systems (e.g., RNA, proteins, lipids, and metabolites), integrating proteomic with other omics datasets is gaining traction (Nesvizhskii, 2014; Miranda-CasoLuengo et al., 2016; Cambiaghi et al., 2018; Zhou et al., 2018; Xu et al., 2020). Adopting such approaches can help answer many questions about disease processes as well as the normal functions they disrupt. Such insights cannot be derived by a single method. Depending on the types of omics data that are being integrated, different challenges may be faced and different new tools may be required. For example, in comparing transcriptomics and proteomics datasets, one main issue is the mapping between proteins and genes. Databases, such as BioMart (Smedley et al., 2009) and Uniprot (Magrane and Consortium, 2011) map proteins to transcriptomic/genomic identifiers, but discrepancies remain as a result of error propagation from legacy issues during automated data integration (Kumar and Mann, 2009). After data are collected for various species, the next important step in the understanding of how pathogens hijack host systems. This will involve comparing similarities and differences across diseases, pathogens, and hosts (for example, see Shah et al., 2015; Gordon et al., 2020). These efforts will help glean comparative insights about disease progression patterns, and guide the design of pathogen-specific as well as pan-pathogen therapies.

## Author Contributions

MR wrote all the R scripts. MR and AG wrote the text and made the figures. AG provided the feedback to MR. MB supervised and supported MR. All authors read and approved the manuscript.

## Conflict of Interest

The authors declare that the research was conducted in the absence of any commercial or financial relationships that could be construed as a potential conflict of interest.
